# Social wayfinding in virtual reality: Navigational decisions and eye movements in a dynamic environment

**DOI:** 10.1167/jov.26.4.2

**Published:** 2026-04-01

**Authors:** Jakub Suchojad, Samuel S. Sohn, Michelle Shlivko, Jacob Feldman, Karin Stromswold

**Affiliations:** 1 Department of Psychology, Rutgers University-New Brunswick, New Brunswick, NJ, USA; 2 RU Center for Cognitive Science, Piscataway, NJ, USA

**Keywords:** social wayfinding, eye gaze, navigation

## Abstract

*Social wayfinding* refers to the process of navigating in the presence of other people. Social wayfinding entails a complex series of interrelated decisions, such as how closely to approach people and when to pass them. In this paper we report two virtual reality experiments that investigate social wayfinding in a complex, dynamic task. In these experiments, participants physically walked from one end of a simulated train station waiting room to the other, avoiding static obstacles (e.g., benches, seated and standing people) and dynamic obstacles (two rows of people walking perpendicularly to the participant’s path). We model the task as a hierarchical combination of local subgoals (e.g., when and where to pass people) and a global goal (which gate to navigate toward). Although eye movements are difficult to analyze in such a dynamic task, they prove to be particularly revealing about how participants combined these local and global goals efficiently in real time. Overall, the results suggest that participants rapidly deploy a flexible combination of local and global decision strategies to navigate crowded environments efficiently.

## Introduction

### Social wayfinding

*Social wayfinding* (SW) refers to the cognitive processes involved in navigating in the presence of other people (Dalton, Hölscher, & Montello, 2019). SW is a common task in everyday life, undertaken whenever we move through populated spaces. Imagine, for example, navigating through a crowded train station toward a particular gate. One must arrive at one’s target location via a reasonably efficient path, avoiding both static obstacles (pillars, walls, and benches) and a gauntlet of other people, some of whom may be navigating themselves. Human wayfinders treat other humans somewhat differently from other obstacles, respecting their personal space and other proxemic boundaries (Sommer, 1959; Hall, 1966). For example, participants are more likely to pass behind than in front of other people (DeStefani, Stromswold, & Feldman, 2022), particularly avoiding crossing others’ gaze (Nummenmaa, Hyönä, & Hietanen, 2009; Kvetnoy, Suchojad, DeStefani, Stromswold, & Feldman, 2025), and passing through perceived social groups (Zhou, Han, Liang, Hu, & Kuai, 2019). The constraints on planning a path are even more complex when other people are also in motion. As a result, traversing a populated space entails a complex sequence of distinct microdecisions, including in which direction to move, and how quickly; which gaps in the crowd to pass through; and where to look to gain the information required to support all these decisions.

Indeed, in our view SW is a quintessential example of the kind of complex and naturalistic task that eye movements are designed to facilitate, as Eileen Kowler often argued (e.g., Bahcall & Kowler, 2000; Kowler, 2011; Kowler, Rubinstein, Santos, & Wang, 2019). In the experiments reported herein, we study SW partly out of intrinsic interest in this ubiquitous task, and also partly as a case study in the ways that eye movements both guide and are guided by dynamically changing input in the context of meaningful behavioral goals.

### SW in virtual reality (VR)

Studying SW in real-world settings poses enormous methodological challenges, primarily because it is virtually impossible for real human “obstacles” to behave in a controlled and repeatable manner (though some studies have done just that) (Croft & Panchuk, 2018). Much research on SW has relied on non-experimental methods, including immersive storytelling (McKeen, 2011), qualitative surveys (Symonds, 2017), and analyses of CCTV recordings (Johnson & Dana, 2022). However, an increasingly common method for studying wayfinding experimentally is VR, which allows participants to be immersed in a virtual environment that can be parametrically controlled and systematically varied (Bönsch, 2024). Particularly important for the studies presented below, this parametric control can be extended to moving virtual humans. Most VR studies use a head-mounted display (HMD), which provides a sense of agency, immersion, and ecological validity (Baggs, Grabarczyk, & Rucińska, 2024). VR has been used in wayfinding-related studies for more than two decades (Fajen & Warren, 2003; Rothkopf, Ballard, & Hayhoe, 2007), as well as in many other areas of psychology, like perception (Wilson & Soranzo, 2015), clinical studies (Turner & Casey, 2014; Riva, 2022), and social cognition (Yaremych & Persky, 2019; Taylor, Valladares, Siepser, & Yantis, 2020).

In the vast majority of VR studies of wayfinding (e.g., Drury et al., 2009; Sharma et al., 2017; Li, Thrash, Hölscher, & Schinazi, 2019; DeStefani, Stromswold, & Feldman, 2020; Bönsch, Ehret, Rupp, & Kuhlen, 2024), participants do not physically move, but rather navigate virtually through space using a combination of keyboard or joystick commands. Although these choices may represent necessary concessions to practical concerns (such as a lack of space to accommodate naturally locomoting subjects), they nonetheless limit the ecological validity of the work. Studies of ambulating (freely walking) subjects are comparatively rare, although recently there have been some exceptions, including studies of social effects (Duverné et al., 2020; Raimbaud et al., 2023), collision avoidance (Berton, Olivier, Bruneau, Hoyet, & Pettré, 2019; Bourgaize, Cinelli, Raimbaud, Hoyet, & Olivier, 2024), and navigation (Meerhoff, Bruneau, Vu, Olivier, & Pettré, 2018).

In the studies we present, we aimed to build on this approach and make it more complex, general, and realistic. Our experimental participants are completely untethered (wireless), because standalone applications deployed fully on VR HMDs can create an even more immersive, naturalistic experience. Our participants physically walk through a real space (an empty room slightly larger than the virtual space) and control their movement through the environment via ordinary head and body movements recorded by the HMD. This setup greatly increases the naturalism of the participant’s behavior (Riecke et al., 2010; Roupé, Bosch-Sijtsema, & Johansson, 2014). VR admittedly has a number of limitations relative to real life (e.g., reduced field of view, limited auditory feedback for sound localization), and the practical difficulties that arise when using ambulating participants (e.g., the need for a large empty room in which to conduct the experiments) can be challenging. However, in light of its many benefits (the immersive and modifiable environment, the availability of untethered HMDs, and the possibility of collecting eye movements through the headset), VR is currently “the best game in town” when it comes to studying SW experimentally.

Previous work in our lab has shown that VR can elicit strong social effects. For example, in DeStefani, Stromswold, and Feldman (2022), we used VR to study how people pass by a single person. In these studies, a single static obstacle, either a virtual human (“agent”) or a virtual inanimate object (an empty chair) facing one of the four cardinal directions, was placed in the middle of an otherwise empty room. Participants had to reach the door on the other side of the room, passing around the obstacle. The results revealed a number of notably social effects. When the obstacle was a (human) agent, facing to the left or right, participants were strongly biased to pass *behind* them, as if to avoid crossing their gaze (De Stefani, 2023). In a study carried out during the COVID-19 pandemic, participants kept a significantly greater distance from unmasked than masked agents (DeStefani et al., 2023). These findings can be interpreted in terms of modifications to personal space (Amaoka, Laga, & Nakajima, 2009; Sanz, Olivier, Bruder, Pettré, & Lécuyer, 2015), the shape of which appears to be sensitive both to the agent’s facing direction and also to more cognitive concerns, such as the likelihood of contagion. The induced personal space also appears to be elliptical and off-centered, extending much further in front of the agent than behind them. This modified personal space strongly influences participants’ navigational choices, for example, biasing them to cross behind other agents. These studies demonstrate that virtual simulations can elicit strong social effects, even in simple tasks with entirely static human (and non-human) obstacles. In the studies reported below, we introduce virtual *moving* agents, making the SW context substantially richer and more naturalistic.

### The role of eye movements in natural tasks

In her work, Kowler often emphasized the role of meaningful contexts and goals in guiding behavior. As she argued, eye movements help to guide, and are in turn guided by, basic cognitive mechanisms of prediction, learning, and attention. Specific behavioral outcomes such as fixations (focused gaze) and saccades (scanning gaze) all further the underlying aim of effective behavior (Kowler, 2011). Bahcall and Kowler (2000) showed that scanning behavior is adaptively modified to optimize perceptual accuracy, and Melcher and Kowler (2001) showed how saccades are apparently programmed to aid memory. In more recent work, Kowler and her students emphasized the role of prediction in smooth pursuit (Kowler et al., 2019) and the role of smooth pursuit in dynamic tasks (Wang & Kowler, 2021). A common element in all her work was a question she frequently posed: what are participants actually optimizing? In natural tasks, the answer to this question is not always obvious.

Beyond Kowler’s work, there have been a number of studies of eye movements during natural tasks, such as making tea (Land, Mennie, & Rusted, 1999), hand washing (Pelz & Canosa, 2001), assembling toys (Steinman, Menezes, Herst, Jenkin, & Harris, 2006), and pitching tents (Sullivan, Ludwig, Damen, Mayol-Cuevas, & Gilchrist, 2021). Of particular relevance to our task are studies of (non-social) navigation (Hayhoe & Ballard, 2005; Rothkopf, Ballard, & Hayhoe, 2007; Matthis & Hayhoe, 2015; Matthis, Yates, & Hayhoe, 2018; Zhang et al., 2018), sequential tasks (Ballard, Hayhoe, Li, & Whitehead, 1992), and learning over repeated actions (Diaz, Cooper, Rothkopf, & Hayhoe, 2013). In many discussions of early results from our lab (see SW in virtual reality), Kowler remarked that our paradigm was well-suited to studying eye movements during a naturalistic task, especially those that involve dynamically changing objects—an aspect sorely missing from the literature because of the technical complexity involved in constructing such a task.

A number of recent technical advances have made it practical to run a naturalistic eye tracking task in VR and to interpret the resulting data. Among these are improved methods for graphing and interpreting three-dimensional (3D) gaze data (Duchowski et al., 2022), for distinguishing fixations from saccades (Kasneci, Kasneci, Kübler, & Rosenstiel, 2014; Dai, Selesnick, Rizzo, Rucker, & Hudson, 2017), for identifying post-saccadic oscillations and smooth pursuit (Lüken, Kucharskỳ, & Visser, 2022), and for understanding other characteristics like focal and ambient gaze (Krejtz, Duchowski, Krejtz, Szarkowska, & Kopacz, 2016) and gaze entropy (Shiferaw, Downey, & Crewther, 2019). Additionally, VR-friendly methods for inferring cognitive load from pupillometric data are now available (Duchowski, Krejtz, Gehrer, Bafna, & Bækgaard, 2020), continuing a tradition long present in cognitive science (Kahneman & Beatty, 1966; Ekin, Krejtz, Duarte, Duchowski, & Krejtz, 2025). In the analyses below, we use these methods to disentangle the complex combination of goals and subgoals that a natural task like SW entails.

### Motivations for the current study

Our study has several interrelated motivations.

First, we wanted to expand the complexity, naturalism, and immersion of the SW experimental paradigm. Our simulated environment is a virtual train station, replete with multiple virtual people (agents), some sitting or standing in natural poses and others walking through the environment. The dynamic quality greatly complicates both the participant’s task as well as the ensuing data analysis, but which we would argue much more closely approximates the realities of everyday cognition.

Second, we wanted to study how decision-making in such a complex and dynamic task unfolds over time. As discussed below, in SW tasks (and, as Rothkopf, Ballard, and Hayhoe [2007] argue, in almost any real-world task), the participant does not have a single unified goal, but rather has a combination of global goals (e.g., to reach a particular location) and more immediate subgoals (e.g., to pass a through a particular gap in the crowd). We wanted to study how this hierarchical task structure is reflected in our participants’ behavior and eye movements.

Finally, we wanted to push the envelope in the interpretation of eye movements in naturalistic settings. With some notable exceptions (e.g., Rothkopf, Ballard, & Hayhoe, 2007; Rothkopf & Ballard, 2009; Zhang et al., 2018; Raimbaud et al., 2023), the vast majority of the literature on eye movements involves static stimuli and simple behavioral goals, such as looking at or searching for a given target. But in a complex hierarchical task like SW, there is no single well-defined visual target, nor is there a single unified behavioral goal. Rather there is a multiplicity of subgoals that translates into a complex sequence of behaviors and gaze directions (Stuart et al., 2019). Untangling the resulting data is a substantial challenge. Hence in addition to deepening our understanding of SW, we also aimed to advance the state of the art in the interpretation of dynamic behavioral data and eye movement data.

## Methods

We aimed to create a task that the participants found natural and immersive, but that was also structured enough to allow systematic analysis. To this end, we created a task in which the participant had to navigate through a crowded train station waiting room while under time pressure, crossing two rows of moving people along the way. The sequence of rows serve as subgoals which give the task a hierarchical structure (cf., Botvinick, 2012; Eppe et al., 2022, with both a main goal (to reach the target gate) and a number of subgoals (to pass through successive gaps in the crowd). As emphasized by Rothkopf, Ballard, and Hayhoe (2007), the subgoal structure of the task influences the way participants behave and where they look. We discuss this structure in greater detail below and later integrate it into our analysis of participants’ behavior.

### Participants

We recruited naive participants from the Rutgers University community. Experiment 1 had 22 participants (10 self-identified as female, 11 as male, and 1 as genderqueer; mean age = 18.4 years, s.d. = 0.9). Experiment 2 had 20 (new) participants (12 self-identified as female and 8 as male; mean age = 18.6 years, s.d. = 0.88). Across both experiments, the age range was 18 to 22 years. All participants reported normal or corrected-to-normal vision and no visual or mobility disorders. Participants in our pool came from a range of cultural backgrounds, that, combined with the culturally diverse models used for our agents, hopefully diffused any cultural biases present in the data. Participants were compensated with course credits in a psychology class. All research was conducted under the supervision of the Rutgers University Institutional Review Board.

### Virtual environment

**Figure 1.**
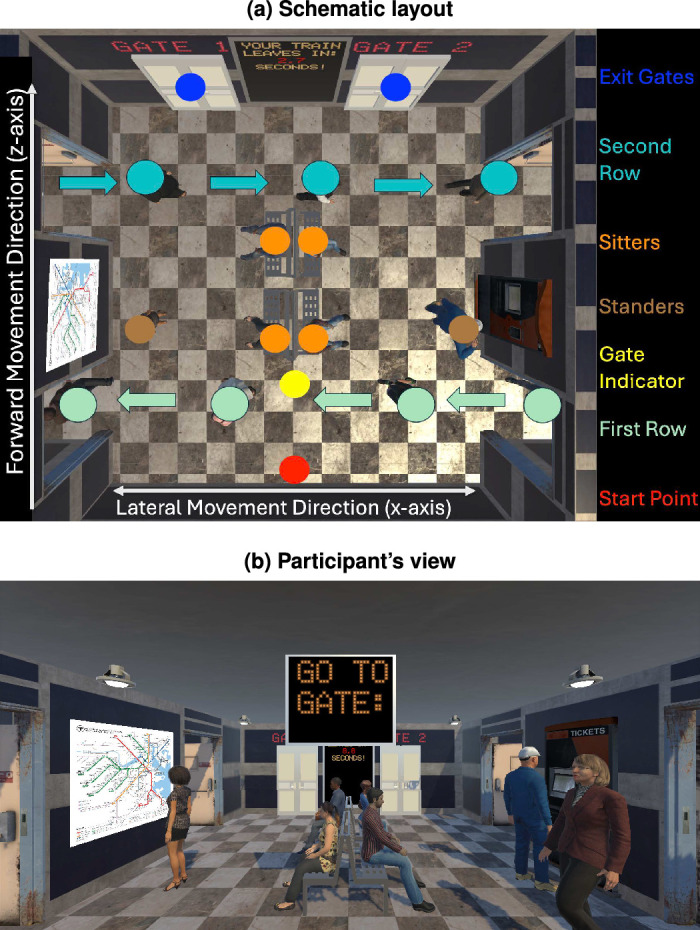
(a) Layout of the virtual train station. Schematic top-down view of the virtual train station. The participant’s starting point is indicated by a red dot (coordinate 0,0), the exit gates are indicated by blue dots (*z*-coordinate = 6). There were two rows of dynamic agents (first row at *z*-coordinate = 1.4 indicated by aqua dots, second row at *z*-coordinate = 4.7 indicated by teal dots). In addition, the environment included static agents sitting on benches (orange dots) and standing before a train map or ticket kiosk (brown dots). (b) Train station from the participant’s point of view: Participant’s view at the beginning of a trial, while waiting for the target gate number to appear.

We chose to simulate a train station waiting room, a place familiar to most of our participants^1^ that often features a wide array of strangers in different positions and orientations. The naturalness and realism of the environment helps to avoid the pitfalls of “gamification”(Hamari, Koivisto, & Sarsa, 2014), while greatly improving immersiveness of the environment compared with our previous work. The layout of the virtual train station is shown in Figure 1, with more details in Table 2.

**Table 1.** Sources of virtual agents and animations.

Agent type	Source
Dynamic agents	Microsoft Rocketbox (Gonzalez-Franco et al., 2020)
Standing agents	Microsoft Rocketbox (Gonzalez-Franco et al., 2020)
Additional agents	Adobe’s Mixamo library
Sitting agents	In-house design using MakeHuman

**Table 2.** Virtual environment specifications.

Category	Element	Specification	Figure reference
Dimensions	Width (*x*-axis)	5 m	Horizontal span
	Length (*z*-axis)	6 m	Vertical span
Key Locations	Starting platform	Coordinates (0,0)	Red marker
	First row of walkers	*z*-position = 1.4	Aqua markers
	Gate Indicator	*z*-position = 1.7	Yellow marker
	Standers	*z*-position = 3	Brown markers
	Benches (sitters)	Center of the room	Orange markers
	Second row of walkers	*z*-position = 4.7	Teal markers
	Exit gates (target and foil)	*z*-position = 6 (far wall)	Blue markers

The environment contained several static objects and a variety of static and moving agents. In the middle of the room were four benches grouped in pairs and positioned back-to-back. This arrangement forced participants to make an initial choice as to whether to proceed left or right. Sitting on these benches were several static agents (“sitters”), whose position and number varied by condition (details in Table 3). Two static agents were located near the side walls, further filling out the space and restricting the area available for navigation. These agents were engaged in natural tasks (viewing a map and buying a ticket) and were programmed to “idle” in place (i.e., to move slightly without changing position). Amaoka, Laga, and Nakajima (2009) identified three qualities that influence the shape of personal space: orientation, relationship, and propensity for evolution in the relationship. Our standers were strangers, positioned with their backs toward the participants, and thus can be presumed not to have implied to the participants the prospect of a social connection, but rather simply that of a human obstacle.

**Table 3.** Design comparison between experiments 1 and 2.

Parameter	Experiment 1	Experiment 2
Deadline	12 seconds per trial	Varied: 8, 10, or 12 seconds
Main manipulation	Bench occupancy: • full benches • sitters on the side of the gate • sitters opposite to the gate	Deadline
Bench condition	Varied	Always fully occupied
Trial distribution	Even split between target gates and sitter conditions	Same as experiment 1
Walkers’ direction	In half the trials, the first row entered from left, and the second from right; vice-versa in other half	Same as experiment 1

Most saliently, two rows of moving agents (walkers) were passing through the room, entering and exiting through side doors, perpendicular to the human participant’s direction of movement. The first row of walkers was closer to the starting position, and the second row was closer to the target gates (see bird’s-eye view in Figure 1). Within each row, the agents walked in single file, alternating men and women, equally spaced (2.5 m apart), and all walking at the same speed (1.3 m/s). The agent models were sourced from Rocketbox (Gonzalez-Franco et al., 2020), including equal numbers of male and female models with a range of apparent cultural identities reflecting the diversity of this dataset. Table 1 gives more details about the sources of the assets used to create the agents.

The number of male and female agents was balanced in each trial. All the moving agents used a motion-capture animation provided in Rocketbox. To address known concerns surrounding both perceived motion (McDonnell, Jörg, McHugh, Newell, & O’Sullivan, 2008; Pražák & O’Sullivan, 2011) and agent variability (McDonnell, Larkin, Hernández, Rudomin, & O’Sullivan, 2009), we left–right reversed every other agent to avoid a “marching” effect where all arms swing in unison. We also avoided repeating any single agent model within a single trial, ensuring that each room comprised a diverse set of unique individuals. All walkers’ *z*-positions were constrained (1.4 m for first row, and 4.7 m for the second row). To avoid any potential confounding effects of gaze engagement or emotional expression (Mousas, Koilias, Anastasiou, Rekabdar, & Anagnostopoulos, 2019; Bönsch, Radke, Ehret, Habel, & Kuhlen, 2020), the agents were programmed to always look straight ahead with a neutral facial expression.

One of the main aims of our studies relative to previous work on navigation in VR was to improve the ecological validity and realism of the task. A central priority was the design of the virtual train station and the virtual humans that populate it. For example, all of the virtual agents populating the space were realistically sized, so that close approaches would yield realistic face-to-face encounters, avoiding any catastrophic breakdowns of immersion. As mentioned, the train station itself featured a map and a ticket machine, as well as ceiling lamps, helping to make it feel like a real-life space. Although the total area of the virtual train station (necessarily no bigger than the real space in which the experiment was conducted) was smaller than a typical train station waiting room, it was big enough to require participants to plan how to traverse it. Thus, although the experiment admittedly did not simulate the entire train station experience, it plausibly recreated the last few meters before reaching the gate. Of course, an experiment in a real train station using eye tracking glasses would be even more ecologically valid, but would lack the precision, parametric control, and repeatability that VR provides. Indeed, most of the analyses presented would have been impossible without the precise knowledge our paradigm affords about the locations and movements of all elements in the virtual scene.

### Experimental procedure

The participant’s task was to traverse from the starting position to one of the two doorways (gates) at the other end of the room. Specifically, participants were instructed, “In each trial, you have to try to catch your train before it leaves the station.” A timer indicating the time remaining was always visible to the participant on the far wall between the two exit gates. To complete the task successfully, participants had to reach their target exit gate within a given deadline (12 seconds in experiment 1, variable in experiment 2), although no feedback was given. The target gate number (1 for left, 2 for right) was indicated 2 seconds after the beginning of each trial on a plainly visible virtual digital sign (gate indicator) hanging from the ceiling about 1.7 m in front of the participant’s starting position (almost directly above the first row of agents). This delay was introduced mostly to allow the walkers in the first row to move far enough across the station that they become a concern for the participant.

Before beginning the experiment, participants completed a tutorial in the experimental VR environment, but with all the agents removed. Participants were allowed to freely explore, and they completed four transitions between trials to ensure they understood the mechanics of the study. After the practice trials, participants received the experimental brief, the HMD was refitted and the eye tracker calibrated, after which the participant began the experimental trials. At the end of the experiment, participants answered demographic questions (e.g., gender, major), and were asked for their impressions about the experiment (e.g., realism, comfort).

Experiment 1 included 2 target gates (left or right) × 2 walker directions (row #1 moving left/row #2 moving right or vice versa) × 3 sitter positions (left, right, or both sides of the benches), resulting in 12 distinct conditions (Table 3). In experiment 2, the sitters were always on both sides of the benches, and the manipulations were 2 target gate sides × 2 walker directions × 3 time conditions (8-, 10-, or 12-second deadline), again resulting in 12 distinct conditions. In both experiments, participants completed 7 repetitions of each condition presented in a fully random order, amounting to 84 trials per experimental session.

### Phase structure and local/global division

**Figure 2.**
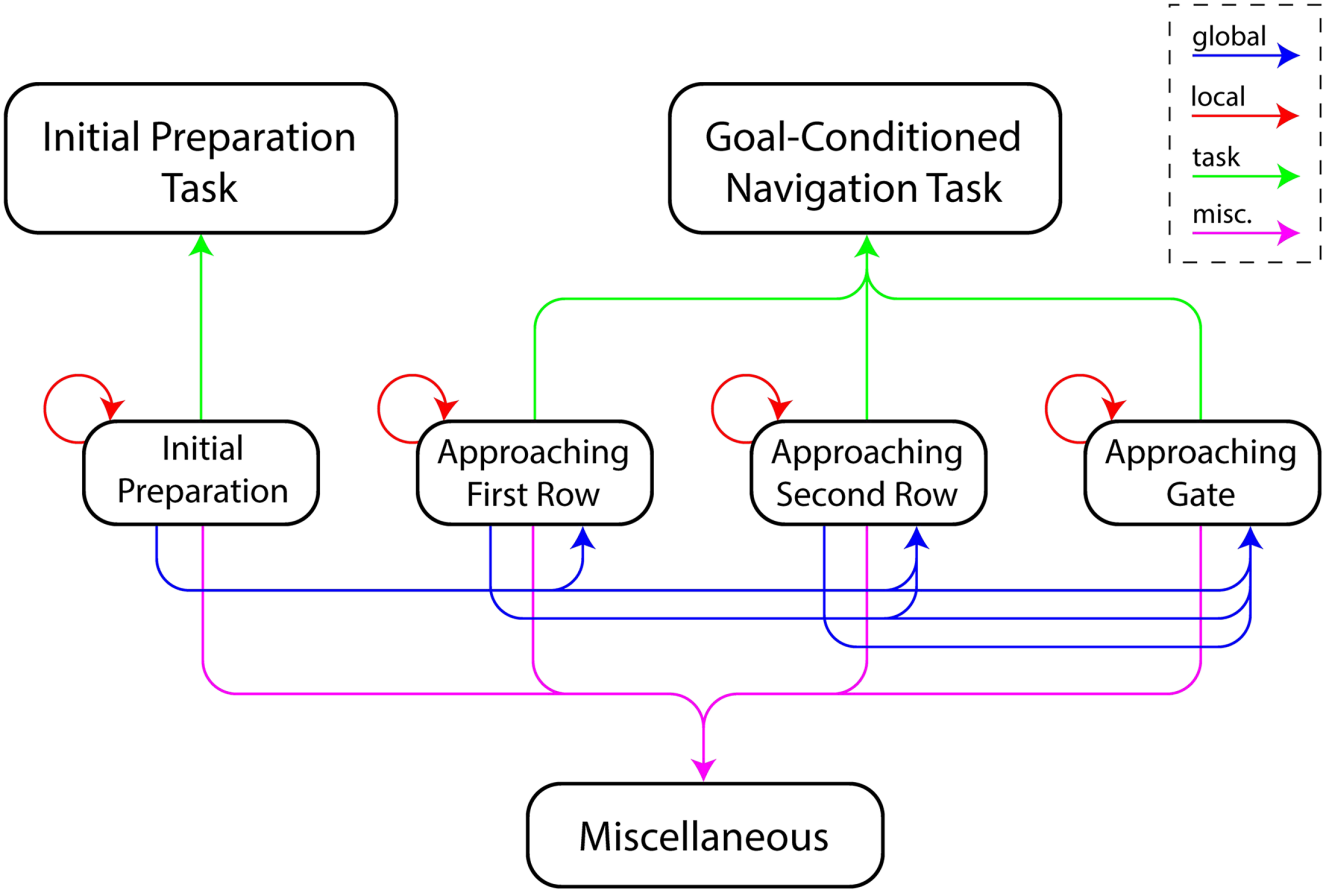
Goal structure of the SW task, showing the overall goal and 4 subgoals (phases). Colored arrows indicate types of eye movements: global/look-ahead (blue), local (red), task-related (green), and miscellaneous (magenta).

As mentioned, the phase structure of the task gives it a hierarchical structure, in which passing through each of the walker rows constituted a subgoal. The goals and subgoals have a dependency structure illustrated by a diagram in Figure 2.

For analytical purposes we divided each trial into four time phases, differentiated by the participant’s immediate goal: 1) initial preparation, 2) approaching first row, 3) approaching second row, and 4) approaching gate (Table 4). Note that the bookend phases initial preparation and approaching gate are different from the middle two. In the initial preparation phase, participants do not yet have the information required to complete the task, so effectively they are just waiting to start. In the approaching gate phase, all of the problem-solving is complete, and participants are simply taking a few last steps toward the exit gate. The middle two phases (approaching first row and approaching second row) are the core of the study, during which participants must actively decide how to pass through the crowd.

**Table 4.** Experimental trial phase structure.

Name	Definition	Sub-task	Attention focus
Initial preparation	Timestamp < 2 s *z*-position < 1.4	Gather required information - wait for the target gate number	*Local:* First row *Global:* Second row, exit gates, standers, sitters, benches *Task related:* Gate indicator, Timer
Approaching first row	Timestamp > 2 s *z*-position < 1.4	Commence navigating and find a gap in the first row located ∼1 m ahead	*Local:* First row, standers, sitters, benches *Global:* Second row, timer, exit gates *Task related:* Gate indicator, timer
Approaching second row	1.4 < *z*-position < 4.7	Find a gap in the second row, located ∼3 m ahead	*Local:* First row, second row, standers, sitters, benches *Global:* Exit gates *Task related*: Timer
Approaching gate	*z*-position > 4.7	Get to the exit gate before the time runs out	*Local:* Exit gate *Global:* N/A *Task related:* Timer

Following the subgoal diagram (Figure 2), we designated specific elements of the environment to be local, global, task related, or miscellaneous. *Local* objects are obstacles that the subject needs to pass imminently (i.e., during the current phase or early in the following phase), such as a walker in an upcoming row. Global objects are obstacles that the subject needs to pass in some future phase, such as a walker in row 2 when the subject has not yet passed row 1. For the purpose of eye movement analysis, global objects correspond roughly to “look-ahead” eye movements (Pelz & Canosa, 2001). Task-related objects are not obstacles, but rather sources of information in the environment, such as the gate indicator sign and the timer. The remaining eye movements, mostly those directed at task-irrelevant environmental structures or falling outside predefined areas of interest, were classified as miscellaneous.

This division into four types of objects, although admittedly somewhat debatable, is very helpful in understanding the wayfinder’s distribution of cognitive resources. As will be seen in the analyses, participants’ attention is divided between immediate concerns such as avoiding local obstacles and longer-horizon planning such as arriving at the correct gate. This division is essential to understand behavior and planning in natural tasks, especially those in which participants are physically moving through the environment, so that the relation between specific objects and the participant’s plans continuously changes.

### Hardware and software

The virtual environment was designed in the Unity Game Engine, editor version 2022.12, using free texture assets available on the Unity Asset Store. Most of the agent models came from the Microsoft Rocketbox repository (Gonzalez-Franco et al., 2020).

The HMD was a Vive Focus 3 with eye tracker add-on. The HMD records 3D location in the virtual space at each frame (approximately 80 Hz) along with the locations of all other objects (dynamic and static) in the VR scene. Eye-tracking data collection was handled by an in-house C# script based on the Vive Wave SDK’s Eye Manager. To ensure full synchronization between the trajectory and eyetracking datasets, we created a simple TimeKeeper script that provided uniform timestamps to all scripts that requested them. The Unity project was built as a standalone Android application deployed to the HMD.

**Figure 3.**
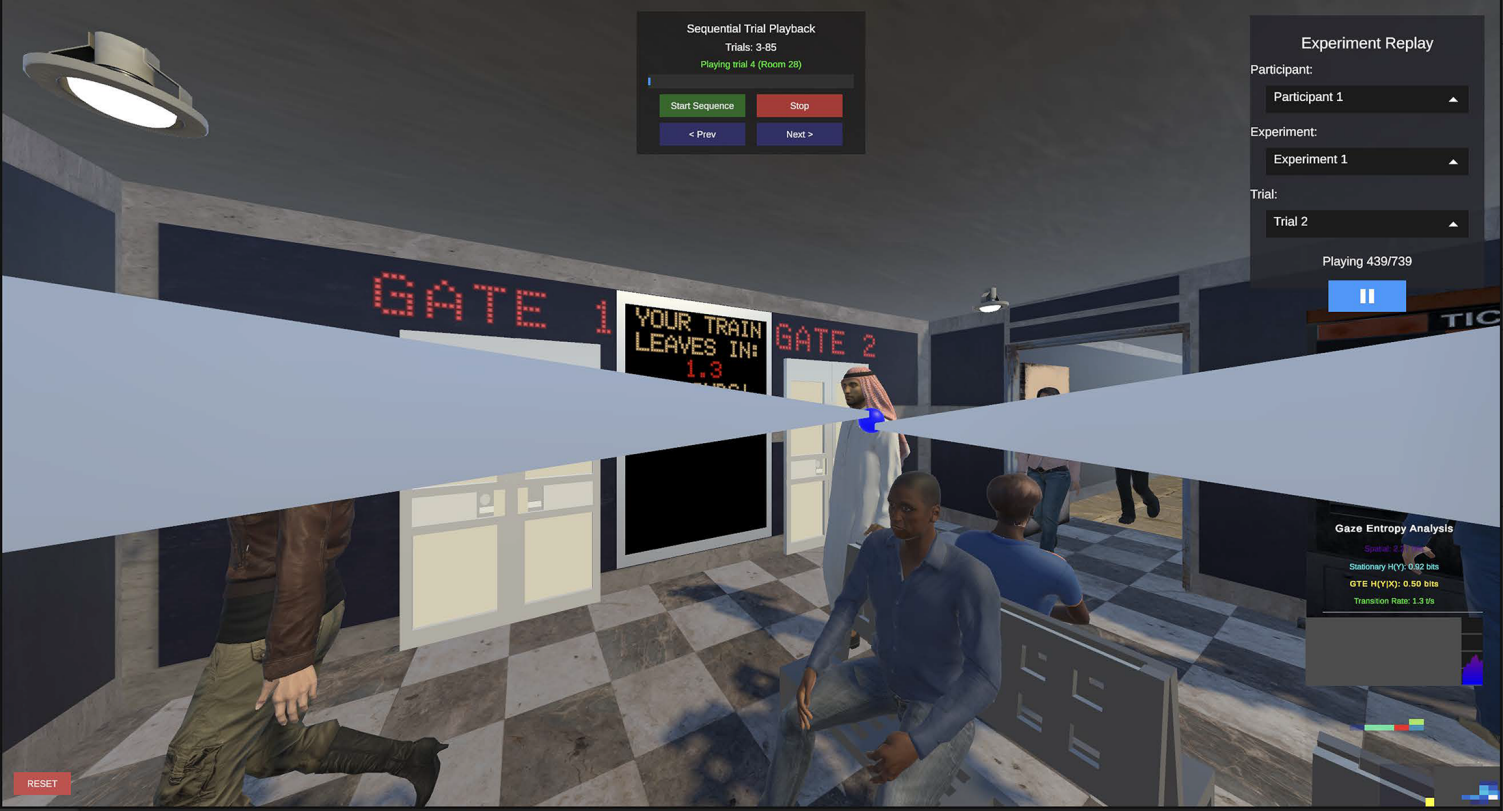
Screenshot of the replay system playing inside of the Unity editor. Subject is fixating on the upcoming virtual agent. A video depicting a few trials is available at this link: https://youtu.be/jo9PYaJ_ZVg.

To determine 3D gaze landing positions in the virtual environment, we used code provided by Duchowski et al. (2022) (augmented by some in-house scripting) to calculate the intersection of the gaze direction vectors from both eyes. As validation, we created a replay system that allowed us to visualize the gaze vectors and their intersection overlaid on the virtual environment viewed by the participant (Figure 3). We also implemented a Savitzky–Golay filter (Savitzky, 1964) based eye movement classifier, which dynamically changes the color of the gaze point depending on whether the eye movement was classified as a fixation or a saccade.

Additionally, we ensured that each object in the virtual train station had a collider, allowing it to be identified as falling along a gaze vector. Comparing these intersections with the locations determined by the vergence method provided a sanity check supporting the gaze location estimates presented below.

## Results

Before we present our results, we stress that we use some common terminology in a relatively loose manner. First, we use the term “attention” broadly to mean cognitive resources directed at an object or task. In most cases, this is reflected in eye, head, or body movements (as measured by the HMD and eye tracker), not *covert* attention (Carrasco, 2011), which we have no way to measure. Second, we use standard eye movement nomenclature (e.g., fixations) to refer to classifications provided by model fitting applied to our raw eye tracking data, which admittedly may not always be accurate. Given that our participants were fully mobile and their heads were not restrained in any way, our measures should be understood as approximations or proxies for categories usually measured under more controlled conditions.

### Data pre-processing

First, we removed trials with a duration of more than 16 seconds (1.62% of trials), because trials of this length usually were the result of technical difficulties, such as failures of the safety boundary or the HMD tracking. In these cases, the technical problems were immediately attended to by the experimenter, who was present in the room, meaning that only individual trials needed to be discarded. The 16-second criterion was chosen so as to allow most good-faith trials while removing all problematic ones. After calculating the statistics presented in Overall performance and Trial duration, we also removed trials where participants went to the wrong gate (1.58% of remaining trials). Finally, we removed data from three participants from experiment 1 and two participants from experiment 2 due to poor eye tracking, yielding a total of 19 and 18 usable participants in experiment 1 and experiment 2 respectively. Additionally, in path shapes analyses (Path shape), we excluded 107 “hooked trials” (for an explanation, see the last paragraph of Path shape). All statistical analyses were computed using R (R Core Team, 2025). All models included participant ID as a random variable and were fitted as full Bayesian regression models using the brms R package (Bürkner, 2017). All models used Gaussian error distributions, which given the sample sizes is usually a reasonable approximation.

### Overall performance

We defined the success in a trial as reaching the target gate within the deadline. A 1-second grace period was added to account for trials where a clear attempt was made to succeed, and the deadline miss was minimal. At the expiration of the deadline, the trials continued while “Your train has left the station” was displayed on the timer board.

#### Experiment 1

In experiment 1 (constant 12-second deadline), the overall success rate was 98.8%. We conducted a Bayesian analysis of success rates using hierarchical linear models. We compared a null model (participant random effects only) against a full model that included sitter position effects, using Bayes factors for model comparison. There was strong evidence against the effect of sitters on success (BF_10_ = 0.001), which is not surprising given that the performance in experiment 1 was near ceiling (For post hoc analyses see Table 5 and Figure 26 in Appendix A). This high success rate presumably reflects the fact that humans are such experts at navigation that the 12-second deadline posed virtually no challenge to our participants. One of the goals of the next experiment was to explore the effects of time pressure more systematically.

**Figure 4.**
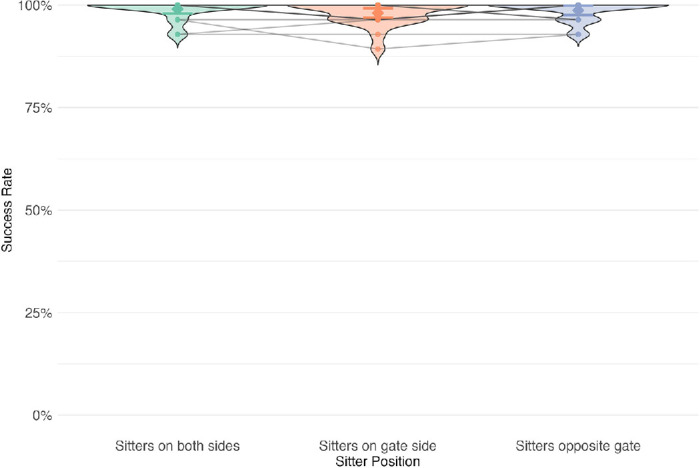
Experiment 1: Effect of sitter position on trial success. In experiment 1, participants performed extremely well. Placement of sitters had no effect on the success (BF_10_ = 0.001). Error bars are 95% credible intervals.

**Figure 5.**
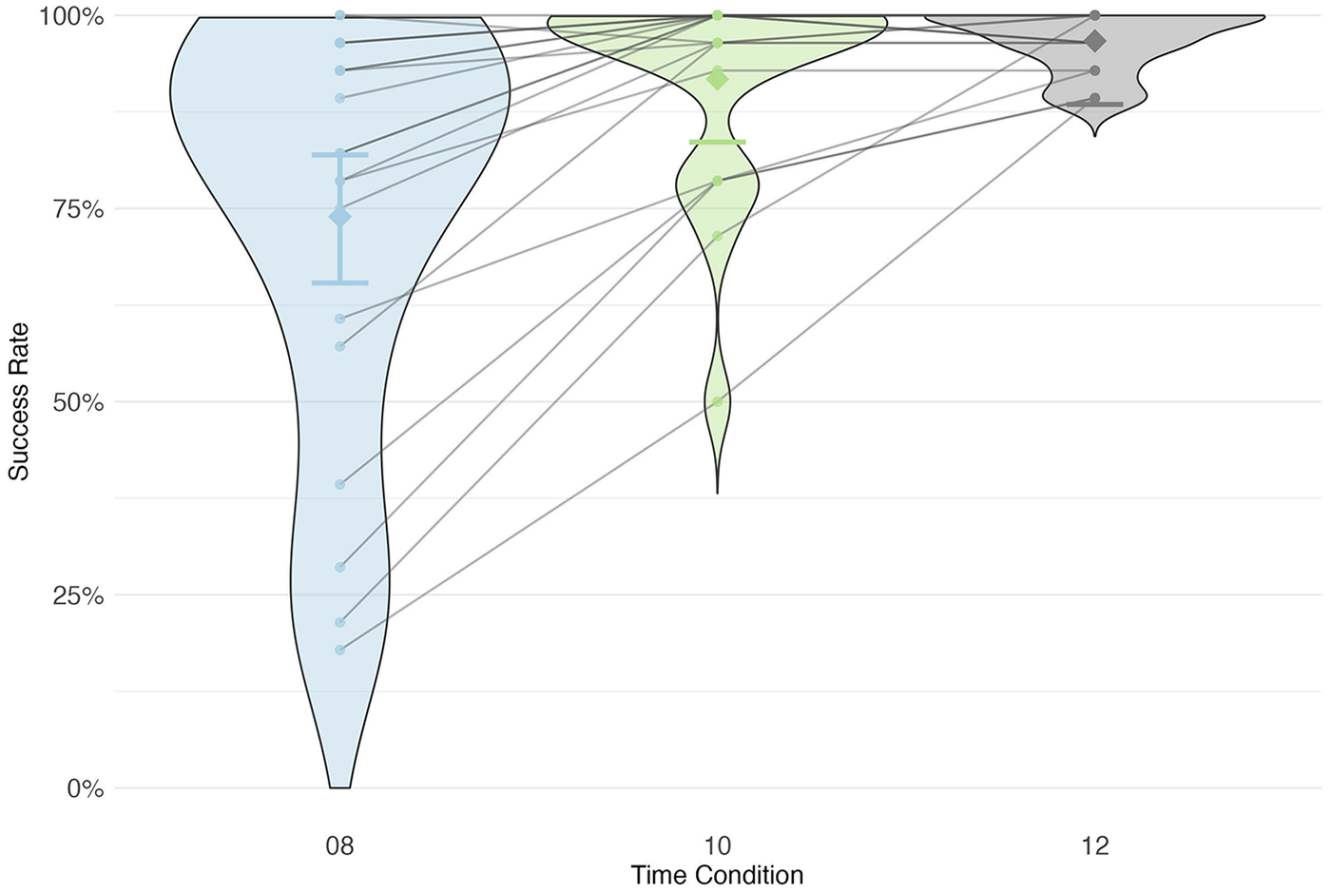
Experiment 2: Effect of deadline condition on success. Overall, success rates increased monotonically with deadline (BF_10_ = 2069).

**Figure 6.**
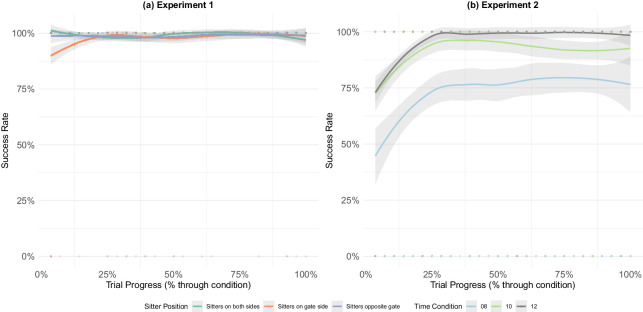
Proportion success (reaching the gate before the deadline) over the course of trials, showing learning effects. (a) Experiment 1. (b) Experiment 2.

**Figure 7.**
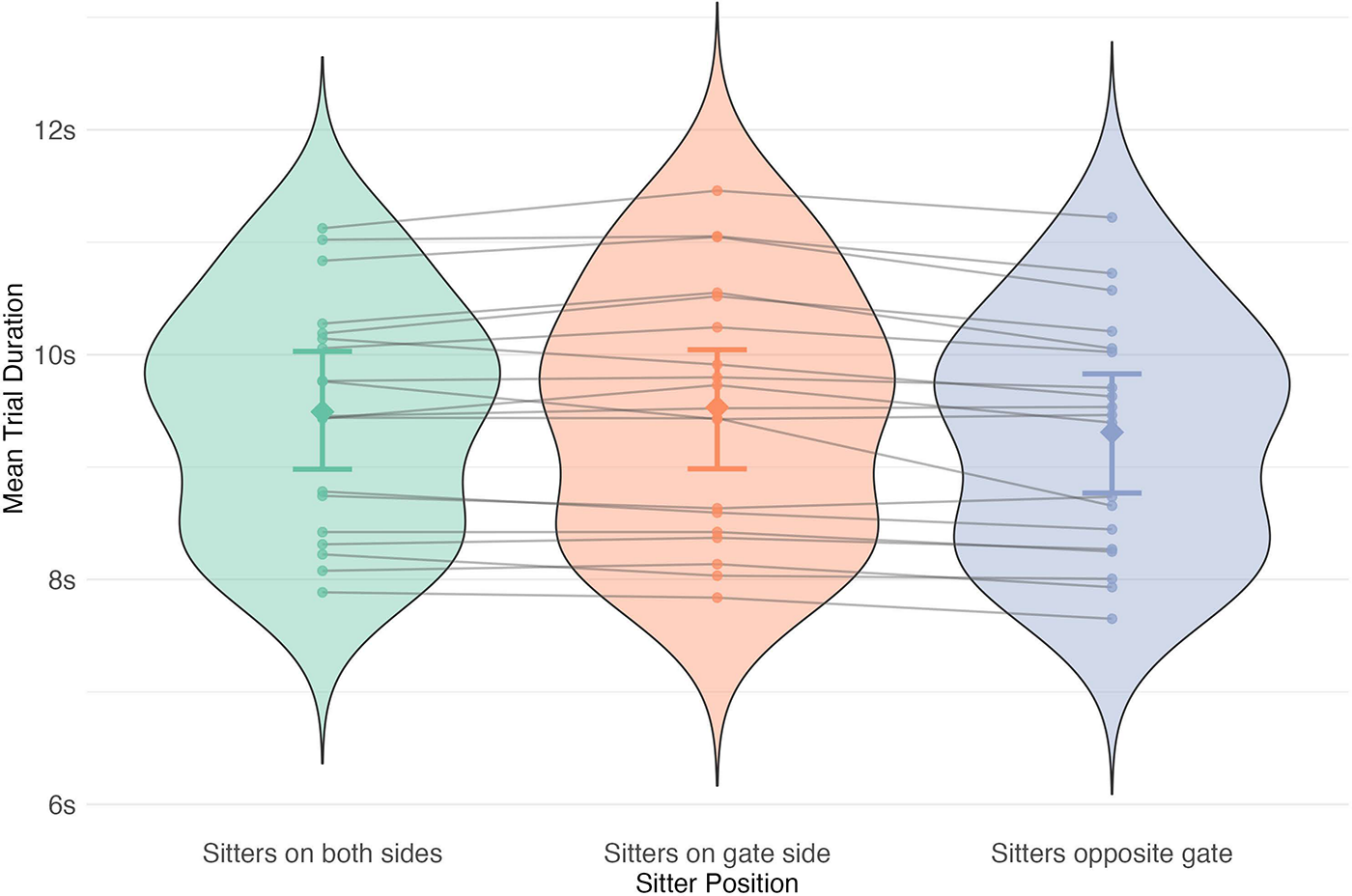
Experiment 1: Effect of sitter position on trial duration. There was a strong effect (BF_10_ = 68) of sitter position. Post hoc analyses revealed that the effect was driven by the ”sitters opposite gate” condition (violet). When the bench on the target gate side was empty, participants on average took less time to move through the station. Error bars are 95% credible intervals.

**Figure 8.**
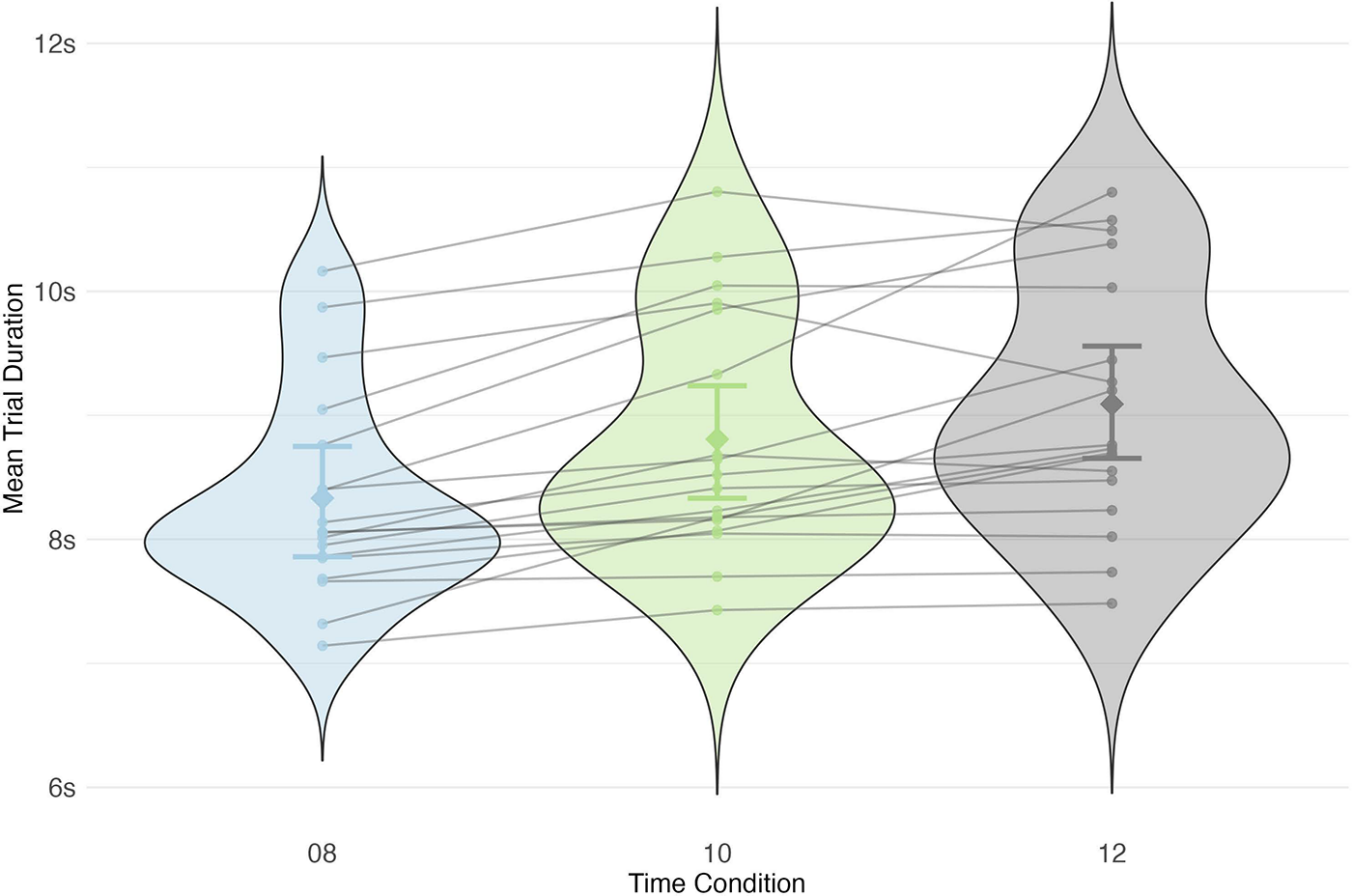
Experiment 2: Effect of deadline condition on trial duration. There was a strong effect of deadline condition (BF_10_ = 7.76 × 10^5^). Trial duration decreased monotonically with the available time. Note that in the 8-second condition (blue) the mean time was longer than the maximum allowed time, while in 10-second (green) and 12-second (gray) conditions, the mean duration was less than the maximum allowed time. This shows that overall people were sensitive to time constraints, but only to an extent. Error bars are 95% credible intervals.

**Figure 9.**
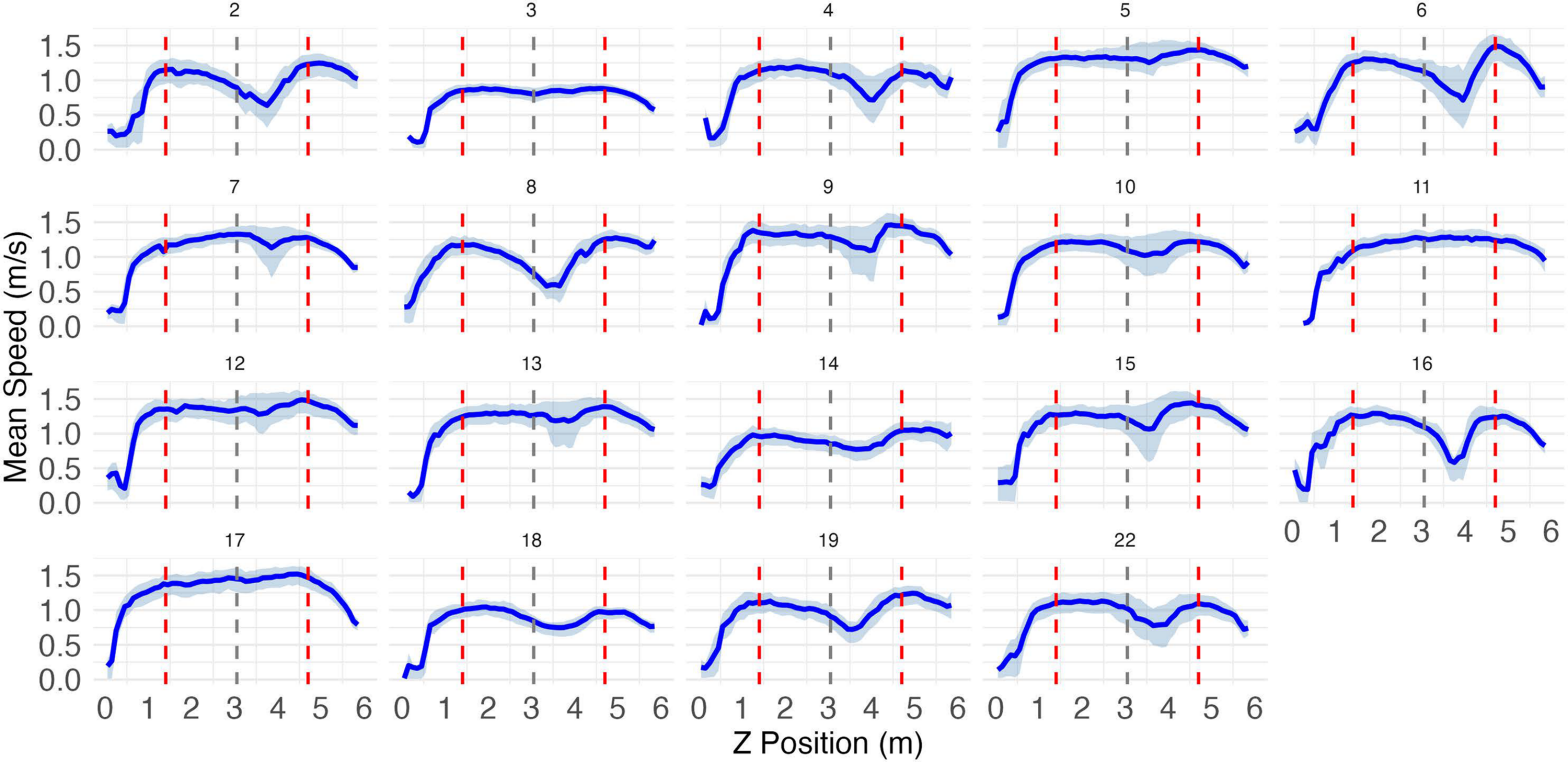
Movement speed profiles as a function of position along the path in experiment 1. Vertical red dashed lines indicate the positions of agent rows (at 1.4 m and 4.7 m), while the gray dashed line marks the midpoint between them (3.05 m). The solid blue line shows mean speed and the light blue ribbons represent the interquartile range (25th–75th percentile) of speeds at each position.

**Figure 10.**
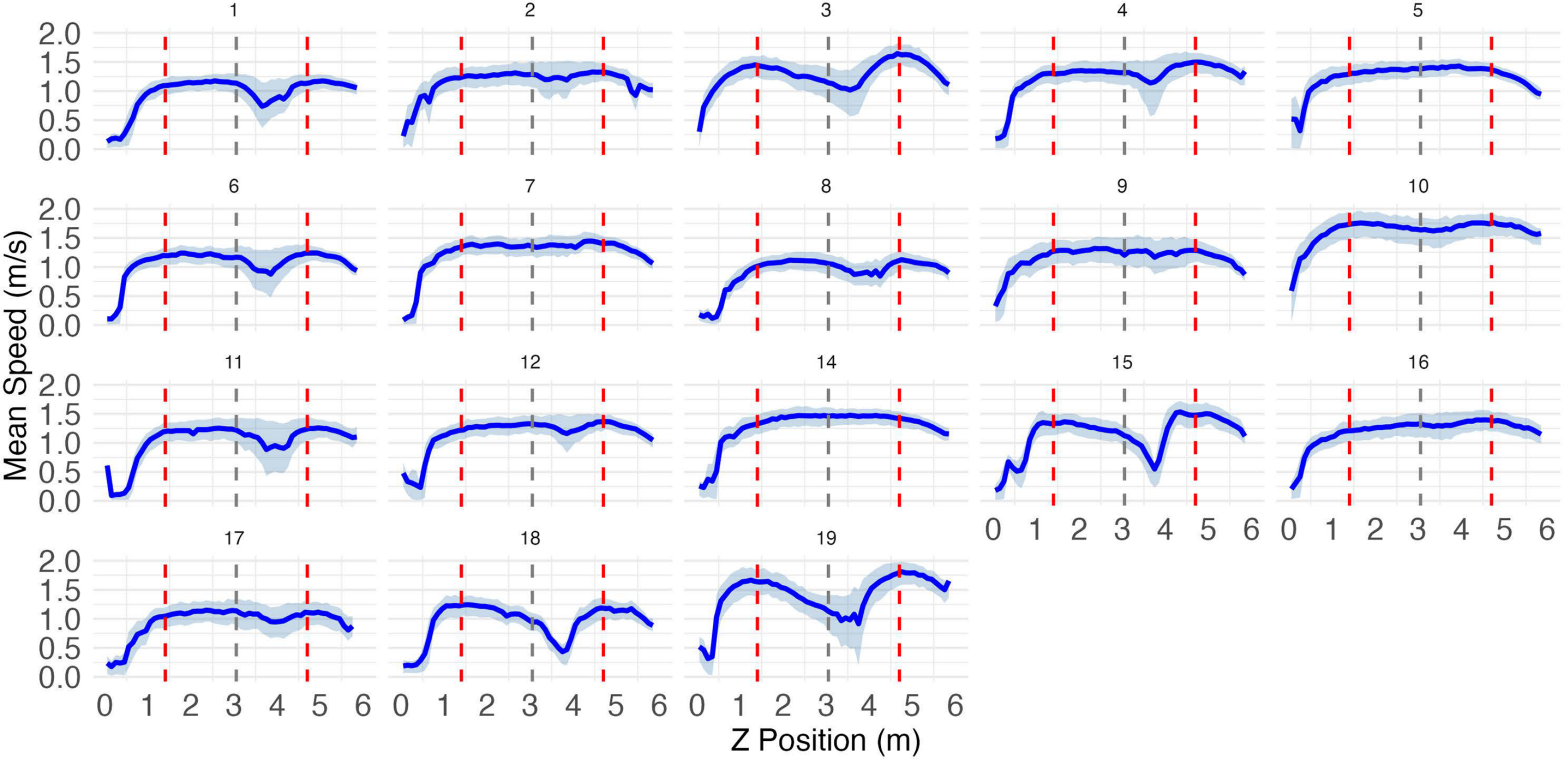
Movement speed profiles as a function of position along the path in experiment 2. Vertical red dashed lines indicate the positions of agent rows (at 1.4 m and 4.7 m), while the gray dashed line marks the midpoint between them (3.05 m). The solid blue line shows mean speed and the light blue ribbons represent the interquartile range (25th–75th percentile) of speeds at each position.

**Figure 11.**
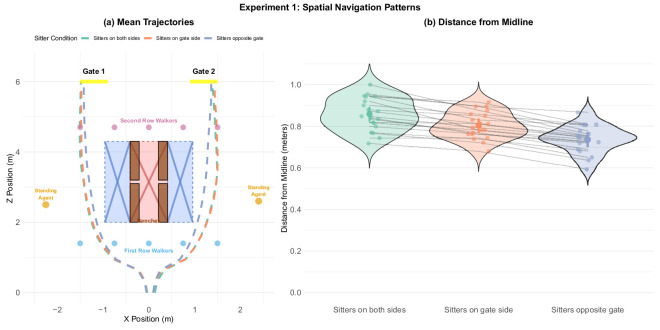
Experiment 1: Midline from distance as a function of sitter position. (a) Mean paths by sitter position depicted to scale. Dashed lines show mean paths per condition. When sitters were on both sides (green) or on the same side as target gate (orange) participants took a wider berth, than when the sitters were located on the benches on the side opposite to the target gate (violet). The red area around the benches symbolizes the space that was inaccessible to the participants due to the benches, while the blue area symbolizes space occupied by extended legs of the sitting agents whenever they were when present. Notice that participants left enough space for the agents’ feet even when they were not present, and shifted even further away when the sitters were present. (b) Effect of sitter position on midline passing distance.

**Figure 12.**
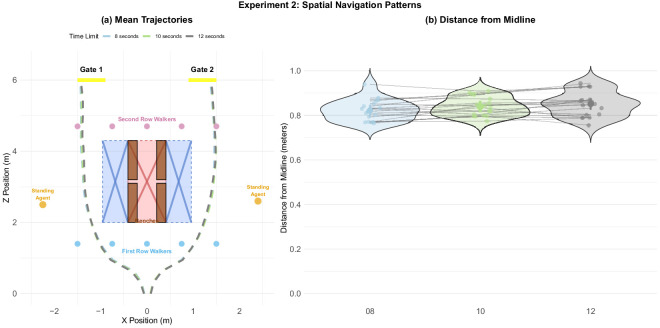
Experiment 2: Distance from midline as a function of deadline condition. (a) Mean paths by deadline condition depicted to scale. Dashed lines show mean paths per condition. The red area around the benches symbolizes the space that was inaccessible to the participants due to the benches, while the blue area symbolizes space occupied by extended legs of the sitting agents whenever they were when present. (b) Effect of time condition on midline passing distance.

**Figure 13.**
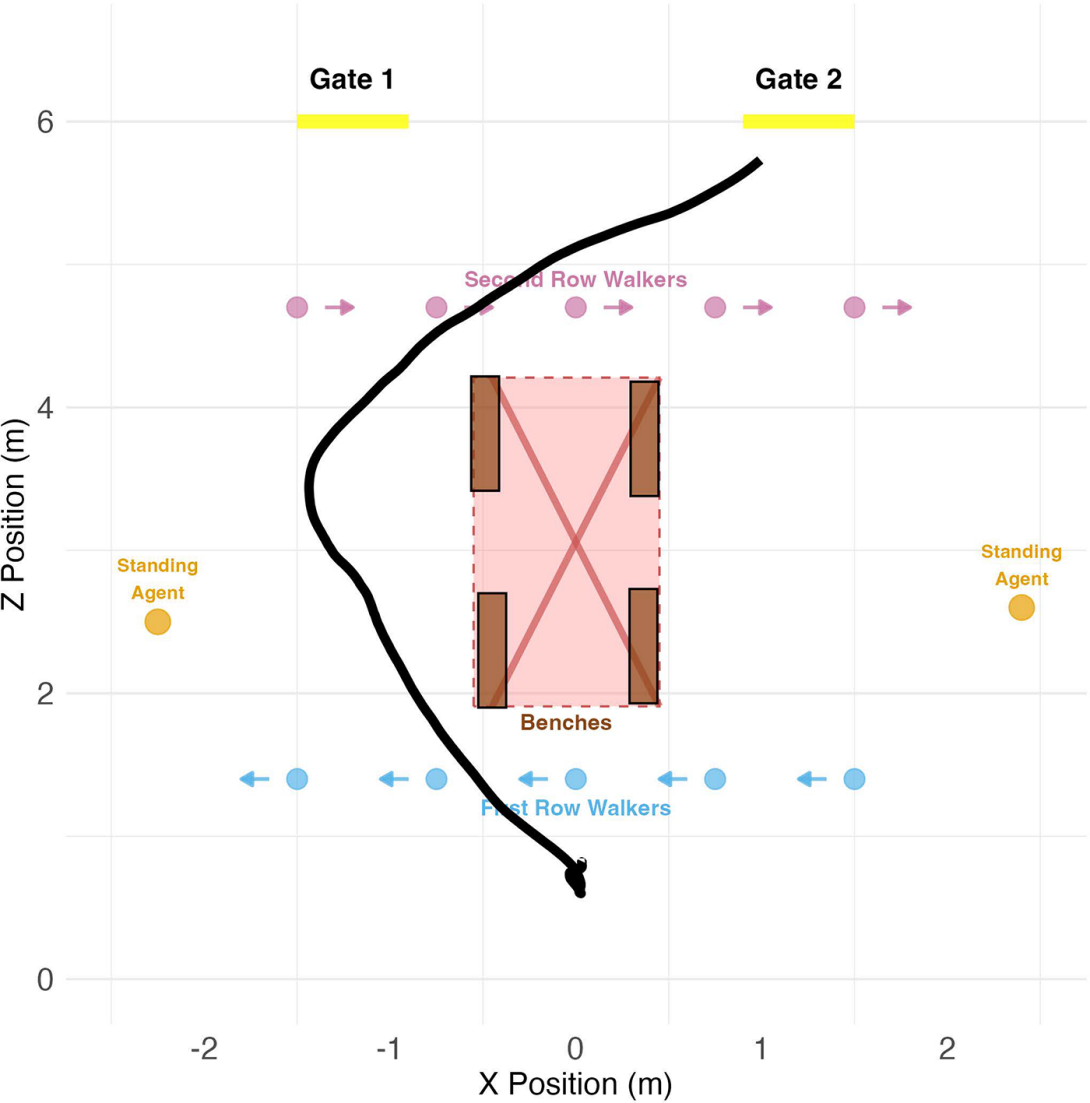
Example of a hooked trial trajectory. Arrows symbolize the usual movement direction during hooked trials, meaning that in most cases, participants followed the flow of the first row.

**Figure 14.**
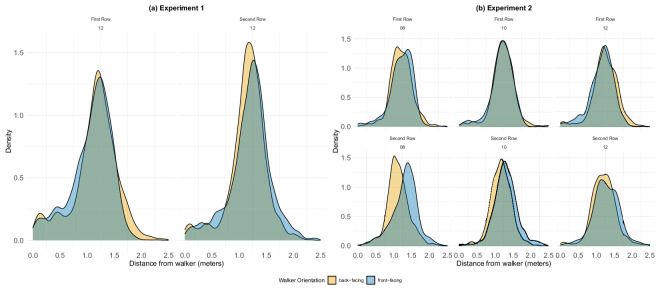
Analysis of the crossing position within a gap. Yellow depicts the mean distance to the front-facing agent, blue depicts the mean distance to the rear-facing agent. (a) Experiment 1: Distribution of distances from the two agents, for rows 1 and 2 from left, respectively. (b) Experiment 2: Distribution of distances from the two agents, for both rows and three deadline conditions (8, 10, and 12 seconds from left, respectively). While in most conditions participants preferred to cross in the middle of the gap, maintaining roughly equal distance from both agents, when under pressure in the 8-seconds condition, they could not afford to wait and moved right behind the passing agent.

**Figure 15.**
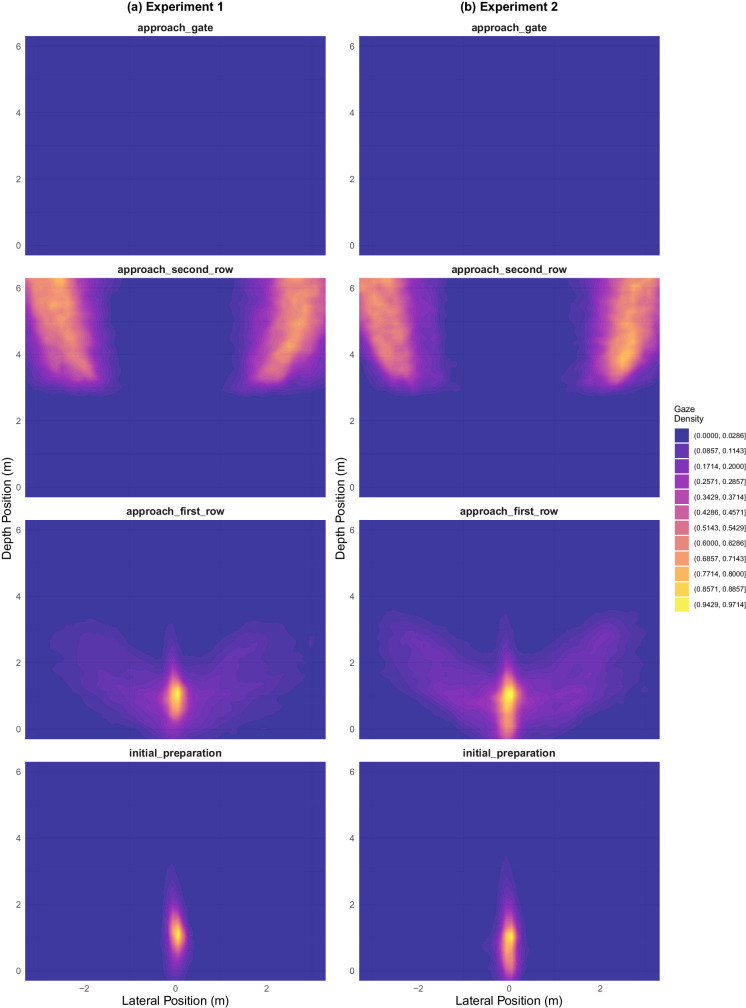
Heatmaps showing probability density of gaze locations. We used a gaussian smoothing filter with a kernel size 0.3 in both directions. (a) Experiment 1. (b) Experiment 2.

**Figure 16.**
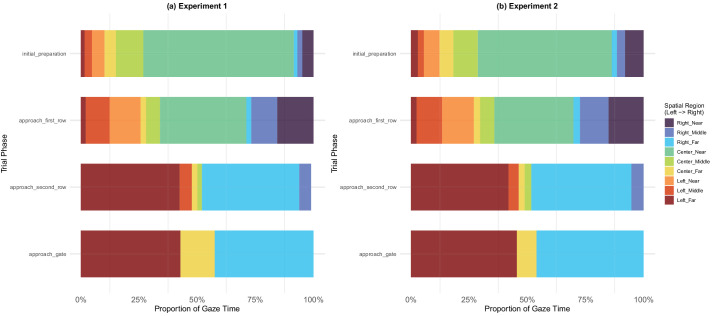
Allocation of gaze broken down by trial phase. (a) Experiment 1. (b) Experiment 2.

**Figure 17.**
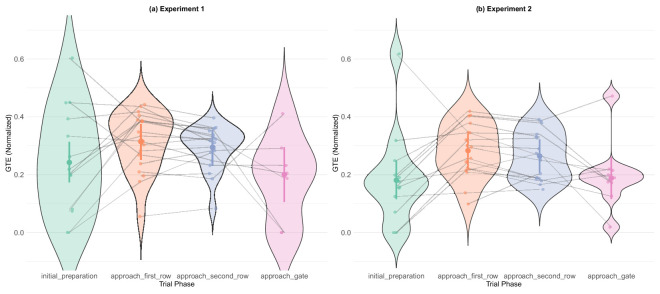
GTE by trial phase. Both experiments show a systematic increase in entropy during active navigation phases (orange and violet) as compared with the two phases where participants were not solving navigational problems (green and pink). (a) Experiment 1. (b) Experiment 2.

**Figure 18.**
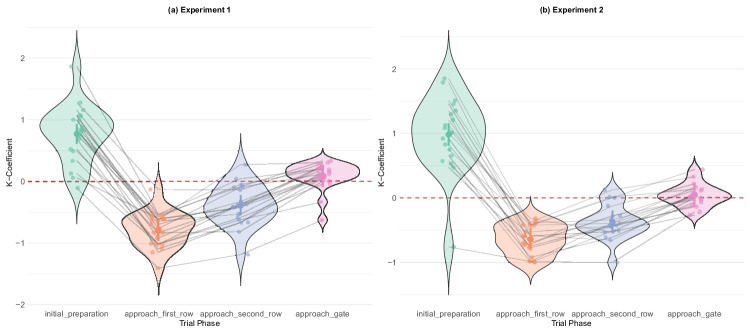
K-coefficient by trial phase. Values above zero indicate focal attention, values below zero indicate ambient attention. The initial preparation phase (green) shows the most focal view, which agrees with the behavioral observation of our participants being mostly focused on the gate indicator sign. (a) Experiment 1. (b) Experiment 2.

**Figure 19.**
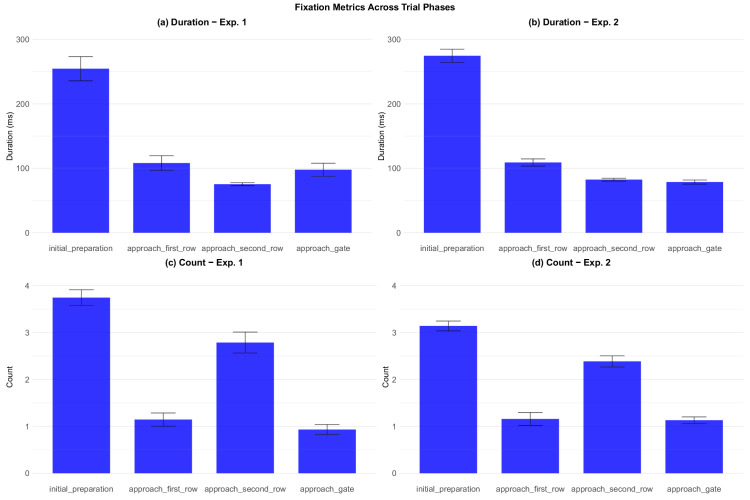
Fixation analysis across experiments and trial phases. Error bars represent ±1 standard error of the mean. (a) Duration - experiment 1 (b) Duration - experiment 2. (c) Count - experiment 1 (d) Count - experiment 2.

**Figure 20.**
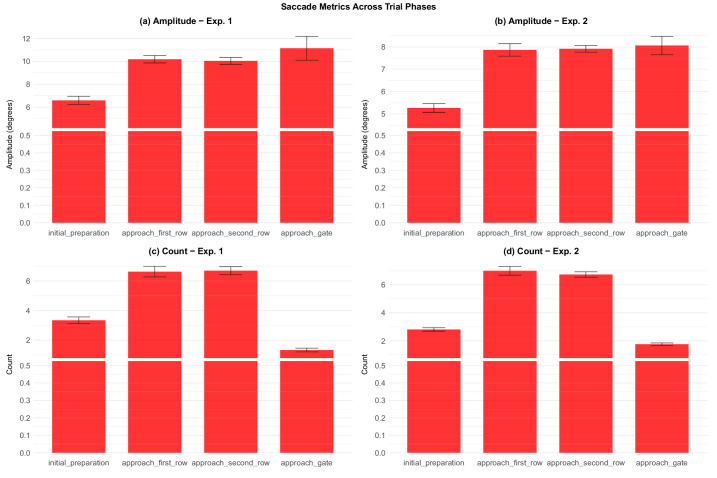
Saccade analysis across experiments and trial phases. Error bars represent ±1 standard error of the mean. Saccade patterns reflect changes in visual search strategy as participants navigate through different phases of the trial. (a) Amplitude - experiment 1 (b) Amplitude - experiment 2. (c) Count - experiment 1 (d) Count - experiment 2.

**Figure 21.**
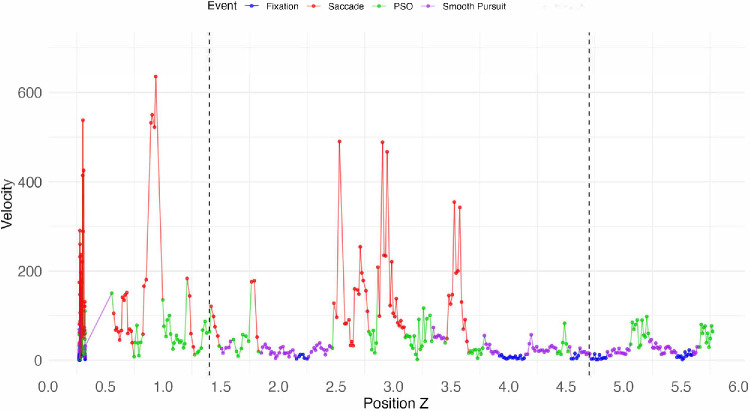
Results of gazeHMM analysis for a single trial, illustrating gaze patterns as a function of the *z*-axis location. Consistent with our expectations, during the navigation phases, there are multiple episodes of smooth pursuit (purple) and fixations (blue) broken up by high velocity saccades (red), followed by PSOs (green) (dashed lines indicate the two rows of walkers).

**Figure 22.**
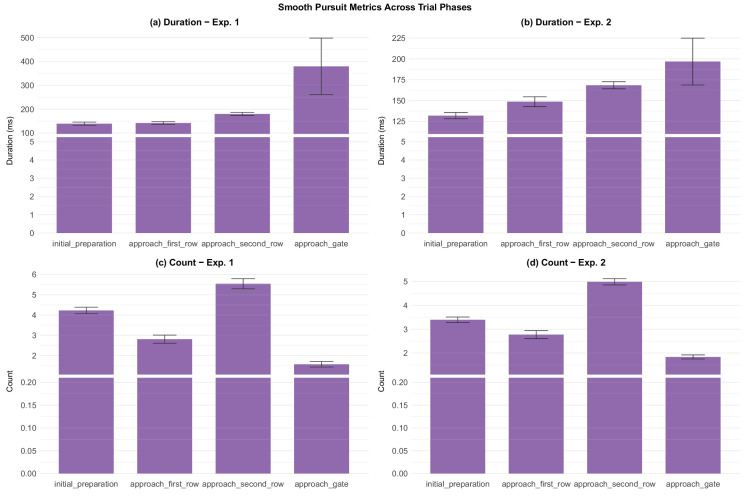
Smooth pursuit analysis across experiments and trial phases. Error bars represent ±1 standard error of the mean. (a) Duration - experiment 1 (b) Duration - experiment 2. (c) Count - experiment 1 (d) Count - experiment 2.

**Figure 23.**
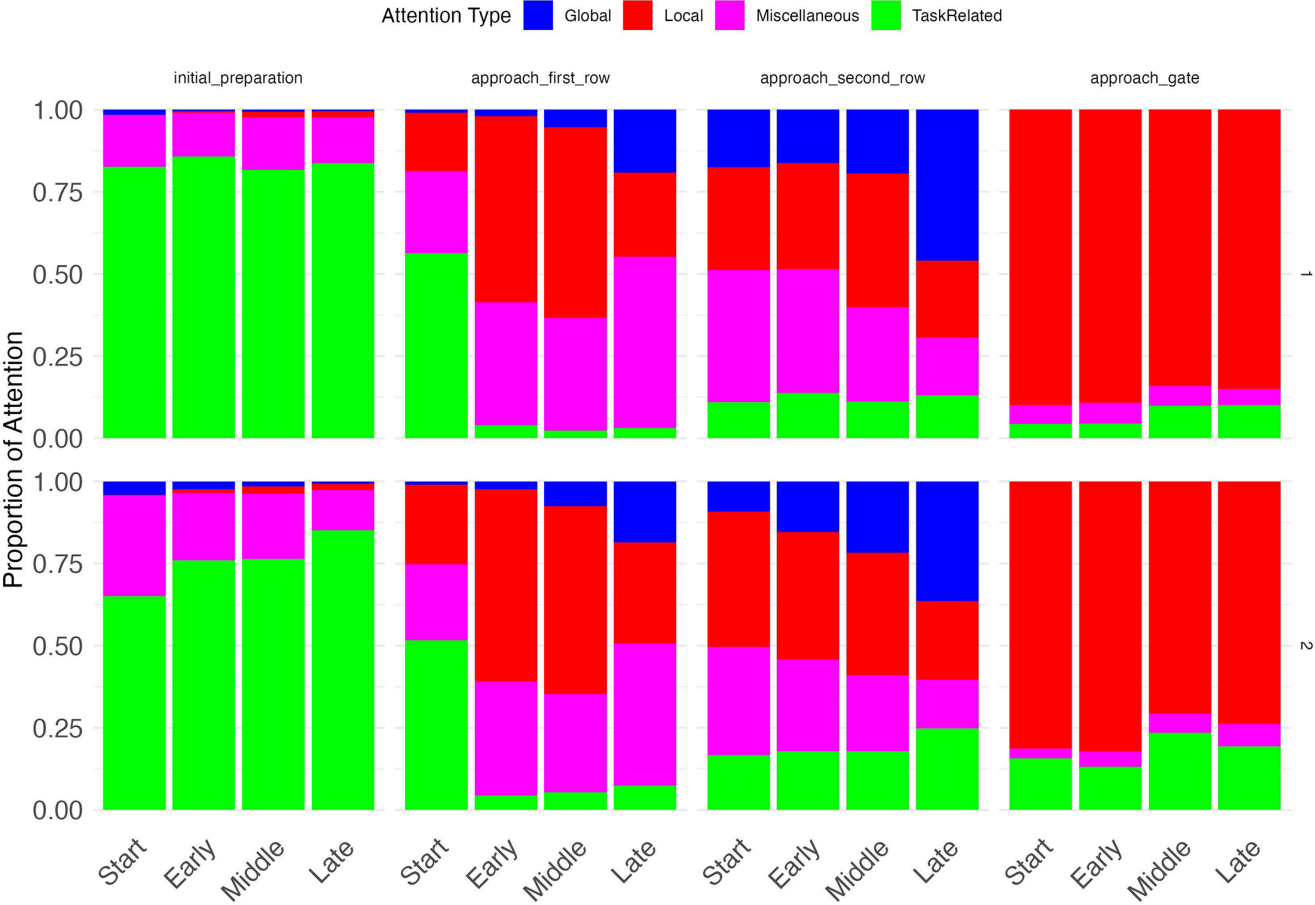
Detailed breakdown of eye movements to different classes of objects. This figure shows mean gaze breakdown for each 1/16th of the trial. At this sub-second scale, we can observe that gaze shift depends on what is currently happening. Although the initial preparation phase is dominated by task related looks to the gate indicator and the timer, followed by highly local (red) period when the subjects were getting ready to cross the first row, followed by a much organized and balanced strategy when getting ready to cross the second row. In the approaching gate phase, we see increased looks toward task related items (green) in experiment 2, which most likely reveals higher number of looks towards the timer owing to the increased time pressure.

**Figure 24.**
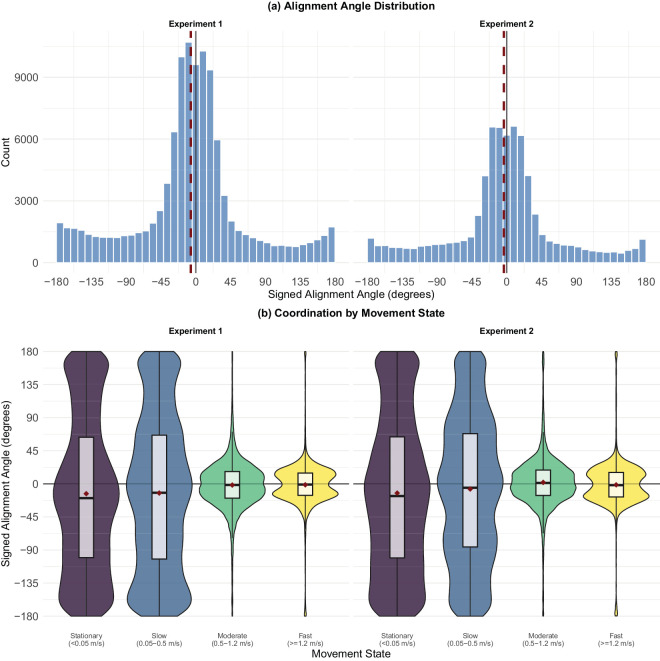
Alignment of movement direction and gaze direction. Positive values indicate looking to the right, and negative values looking to the left. (a) Gaze and movement alignment. (b) Alignment as a function of speed. (b) Shows that high misalignment values are exclusive to when the participant is stationary or moving slowly.

**Figure 25.**
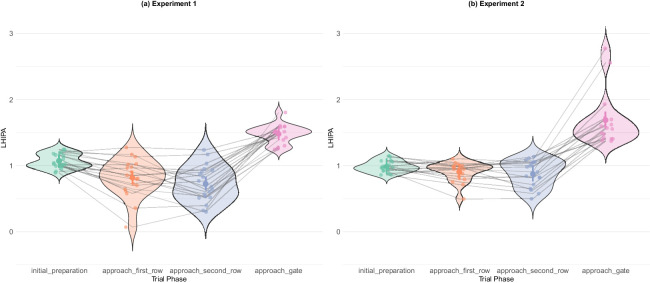
Cognitive load measurements via LHIPA. Trial phase had a strong effect on the LHIPA value in both experiments (experiment 1 BF_10_ = 7.15 × 10^15^; experiment 2 BF_10_ = 5.63 × 10^14^). Navigational phases appear to induce higher load than the preparation phase, whereas the final phase shows a substantial reduction in load. Experiment 2 seems to induce higher load from the beginning of the trial, which stays relatively steady, whereas in experiment 1 the fluctuations appear more robust, with lower load initially, but higher during navigation. (a) Experiment 1. (b) Experiment 2.

#### Experiment 2

**Figure 26.**
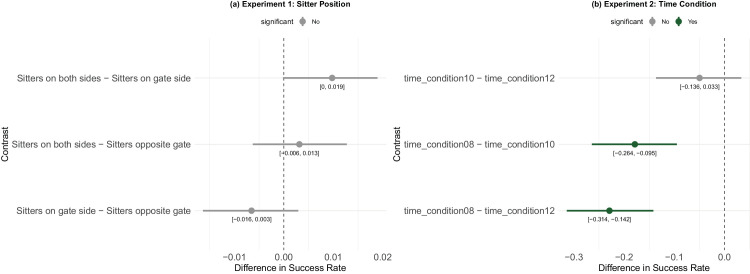
Pairwise comparisons for success rate differences. (a) Experiment 1: Sitter position contrasts. (b) Experiment 2: Time condition contrasts.

In experiment 2 (variable 8-, 10- or 12-second deadline), success rates decreased as the deadline was reduced (Figure 5). A comparison of the null and full Bayesian linear models revealed that there was a strong evidence for an effect of deadline condition (BF_10_ = 2069). Post hoc analysis revealed that the effect was driven by the 8-second condition, in which the success rate was much lower than that in the 10- and 12-second conditions, with only weak evidence for a difference between 10- vs 12-second conditions (see Table 6 and Figure 26 in Appendix A). This analysis suggests that subjects found the 8-second condition fairly difficult.

#### Learning effects

One question that arises naturally in our task is whether (and how) our participants’ performance might change over the course of learning. Figure 6 shows that while in experiment 1 performance was at ceiling almost from the start, in experiment 2 performance took longer to rise to ceiling, presumably because of the more restrictive deadline conditions and the increased cognitive load associated with keeping track of the clock. In the 10- and 12-second deadline conditions, participants took on average about one-quarter of the trials to reach ceiling performance, while in the 8-second condition their performance only asymptoted at about 75%.

### Trial duration

#### Experiment 1

**Figure 27.**
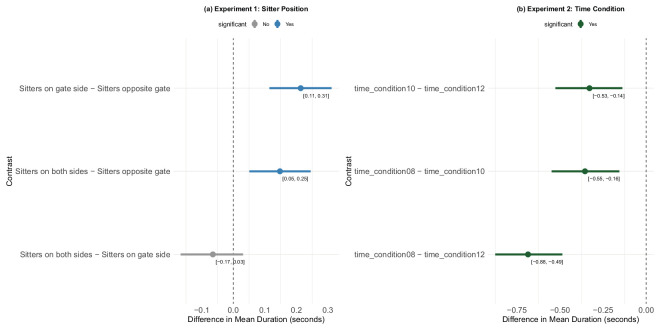
Pairwise comparisons for trial duration differences. (a) Experiment 1: Sitter position contrasts. (b) Experiment 2: Time condition contrasts.

The mean trial duration ranged from 9.32 to 9.54 seconds across conditions. Despite a small absolute difference between the conditions, there was strong evidence for the effect of sitter position on trial duration (BF_10_ = 68) (Figure 7). To verify the large Bayes Factor, we reran the analysis without the Participant ID as a random factor. The resulting BF_10_ = 0.73, confirmed that our analysis results were accurate. Furthermore, post hoc analysis revealed that the effect was driven by the sitters opposite gate condition—the credible intervals did not overlap zero for either comparison, and there was no difference between the sitters on both sides and the sitters on gate side conditions (see Table 7 and Figure 27 in Appendix A).

#### Experiment 2

The mean trial durations ranged from 8.41 to 9.09 seconds across conditions (Figure 8). Bayesian model comparison revealed that there was very strong evidence for the effect of deadline condition on trial duration (BF_10_ = 7.76 × 10^5^). Post hoc analysis revealed very strong evidence for differences between all conditions, with no pairwise credible intervals overlapping zero (see Table 8 and Figure 27 in Appendix A). Note that the difference in mean trial duration between the 8- and 12-second conditions was less than 1 second. Moreover, the mean duration in the 8-second condition was greater than the allowed time, whereas in the 12-second condition it was almost 3 seconds less than the allowed time. These comparisons suggest that participants defaulted to nearly identical timing in all conditions, sufficient to meet the 8-second deadline but excessive for the 10- and 12-second conditions, rather than adjusting on the fly for each condition.

### Movement trajectories

We next analyzed subject’s movement trajectories and velocities.

### Speed

#### Experiment 1

We next analyzed participants’ speed as a function of position on the *z*-axis as they traversed the room. Figure 9 shows mean speed profiles for every participant in experiment 1. While a few participants (e.g., 3, 11, 17) had fairly flat curves, suggesting a ballistic movement with no hesitations, most of the participants exhibited a substantial velocity dip between rows 1 and 2, making their speed profiles bimodal. This slowdown suggests a strategic pause to plan their route through row 2, a sign of local trajectory modification which plays a central role in our analyses.

#### Experiment 2

Most participants in experiment 2 also exhibited a similar slowdown (Figure 10), though not as many as in experiment 1. This suggests that the increased time pressure in experiment 2 forced more participants to carry out their trajectories ballistically, without local modification. As will be discussed, our interpretation is that in the less time-pressured conditions of experiment 1, participants had more leeway to plan socially optimal paths, while in experiment 2 they were forced to prioritize path efficiency.

### Path shape

Next, we analyzed how our experimental manipulations affected the paths taken by the participants.

#### Experiment 1

Most of the participants seemed to follow a smooth, direct, and apparently efficient trajectory from the starting position to the target gate. In experiment 1 (Figure 11a), participants generally maintained a greater distance from the benches if they were filled with sitters.

**Figure 28.**
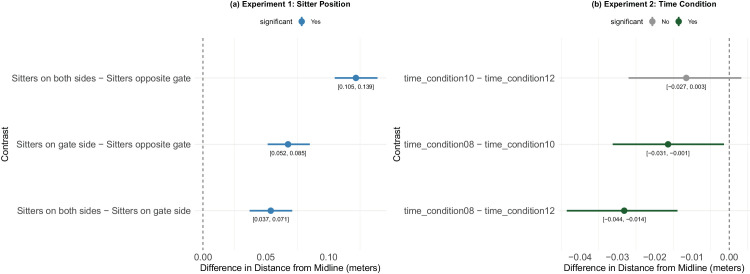
Pairwise comparisons for distance from midline differences. (a) Experiment 1: Sitter position contrasts. (b) Experiment 2: Time condition contrasts.

A Bayesian model comparison showed very strong evidence for the effect of sitter position on distance from midline (BF_10_ = 1.39 × 10^12^), with participants maintaining the greatest distance when sitters were on both sides, followed by sitters on the opposite side (Figure 11b). Post hoc analyses confirmed very strong evidence for all pairwise comparisons, with the largest difference being between sitters on both sides and sitters opposite gate conditions (for post hoc analyses see Table 9 and Figure 28 located in Appendix A).

#### Experiment 2

In experiment 2 (Figure 12a), the trajectories for all three time conditions (8, 10, and 12 seconds) were at first glance nearly identical, with distance from midline ranging from 0.821 m to 0.848 m across conditions. A Bayesian model comparison revealed evidence against an effect of time condition on distance from midline (BF_10_ = 0.19), suggesting that participants did not substantially alter their lateral positioning based on available time (Figure 12b). For post-hoc comparisons (see Table 10 and Figure 28 located in Appendix A).

Taken together, these findings suggest that participants adjusted their paths based on the sitting agents’ positions, consistent with our previous findings about social avoidance, but did not substantially alter their paths due to time pressure.

#### Hooked trajectories

Interestingly, in some trials (6% in experiment 1 and 2.5% in experiment 2) participants adopted a qualitatively different strategy, taking a “hooked” path in which they initially moved toward the non-target gate, and then switched sides late in the trial to arrive at the target gate (Figure 13). Hooked trajectories are physically longer than a direct path, although at least in the less time-pressured, experiment 1, participants who switched sides were still usually able to finish by the 12-second deadline. Switching sides but still finishing on time was more difficult in the 8- and 10-second conditions of experiment 2, which is presumably why hooked trajectories were much less common in experiment 2. Given the rarity of hooked trajectories even in experiment 1, it is unclear if switching sides reflected a performance error or a deliberate alternate strategy.

#### Gap analysis

Recall that participants had to pass between two agents walking in the same direction, meaning that, at the time of crossing, one of the agents was front-facing and the other rear-facing relative to the participant. Therefore, we next examined the location within each inter-agent gap through which participants passed. Figure 14 shows the measured probability distributions of the participant’s passing point for both experiments.

In experiment 1, the mean passing point was approximately midway between front- and rear-facing agents, suggesting that participants aimed to maximize social distance from both agents approximately equally.

In experiment 2, the pattern was similar in the 10- and 12-second conditions, but noticeably different in the more time-pressured 8-second condition. Here participants tended to pass much closer to the rear-facing agent. Our interpretation is that the time pressure induced participants to move rapidly through the gap as soon as the first (rear-facing) agent passed, allowing them to save time. This strategy is especially visible in row 2 (bottom-left in Figure 14b), where the time deadline loomed more heavily. These findings extend our previous results regarding the shape of the personal space surrounding static agents (De Stefani, 2023; DeStefani et al., 2023) to moving agents. As with static agents, the avoidance area surrounding each walker is centered somewhat in front of them, here leading participants to pass closer to the rear of the leading agent than to the face of the following agent.

### Gaze behavior analyses

In this section, we present analyses of the eye movement data collected in the two experiments. These analyses are mainly exploratory, designed to document how participants gathered information over the course of each trial. We used a number of standard analytical techniques drawn from the eye movement literature, but it is important to keep in mind that most of these techniques were developed in the context of traditional experimental tasks involving static subjects looking at two-dimensional computer screens. These techniques do not always translate directly, and one of our goals was to explore how best to apply these techniques to a dynamic, naturalistic task (Orquin & Holmqvist, 2018). Additionally, in these analyses, we do not focus on specific experimental manipulations, but rather on the emerging patterns as a function of our phase-like structure (see Phase structure and local/global division for details.)

#### General gaze patterns

Before performing more specific statistical analyses on gaze data, we first plotted the overall spatial allocation of gaze points (Figure 15). Considering the phase-like substask structure of our paradigm, we expected to find hotspots (regions of high gaze location probability) corresponding with salient locations in each phase, and indeed the figure shows several patterns consistent across both experiments. During the initial preparation phase, gaze was mostly focused on the Gate Indicator, with some warmer spots corresponding to the agents in the first row. In approaching first row, there was a robust elevation in the horizontal spread of gaze, corresponding to the participants’ attempts to identify a passable gap in the first row. During the approaching second row phase, participants scanned widely across the second row of walkers, ranging from their entry point to approximately the point where they passed the target exit gate. After this point, they mostly ignored them, as evidenced by the large gap in the heatmap around the midline of the room. We did not find any distinguishable heat points during the final approach gate phase, presumably because of the very sparse data arising from this very brief phase.

Figure 16 depicts evolution of gaze allocation as a function of phase. The patterns show how the gaze changes from initially centrally focused (due to the gate sign) to being directed almost exclusively toward the extremities of the room, most likely gazing towards the entry point of the second row walkers and the target gate when trying to identify the crossing point.

#### Visual scanning efficiency: Gaze transition entropy and K-coefficient analysis

Next, we explored our participants’ visual scanning patterns. Parsing a naturalistic scene is itself a complex perceptual task, and we hoped to find consistent patterns in how our participants went about it. To that end, we employed two complementary measures: gaze transition entropy (GTE) (Krejtz et al., 2015) and K-coefficient (Krejtz et al., 2016). GTE quantifies the predictability of sequential gaze transitions between spatial locations (Shiferaw, Downey, & Crewther, 2019), whereas the K-coefficient is intended to distinguish between focal and scanning (ambient) gaze. Taken together, these measures should allow us to understand how our participants gaze allocation changed as a function of the subtask they were currently attending.

#### Gaze transition entropy

Gaze coordinates were discretized into a 6 × 12 × 3 spatial grid (216 states), and GTE was calculated using 80-frame rolling windows with Shannon’s conditional entropy, normalized to a 0–1 scale following (Krejtz et al., 2015). Lower GTE values indicate more predictable, efficient scanning patterns, and higher values reflect more random, exploratory patterns. Calculating meaningful values of GTE failed for a number of participants in the initial preparation and approaching gate phases, since the static nature of gaze in these phases resulted in a minimal number of transitions. Nonetheless, we have included these partial results in our analyses, since the brms package (Bürkner, 2017) handles unbalanced models well.

**Figure 29.**
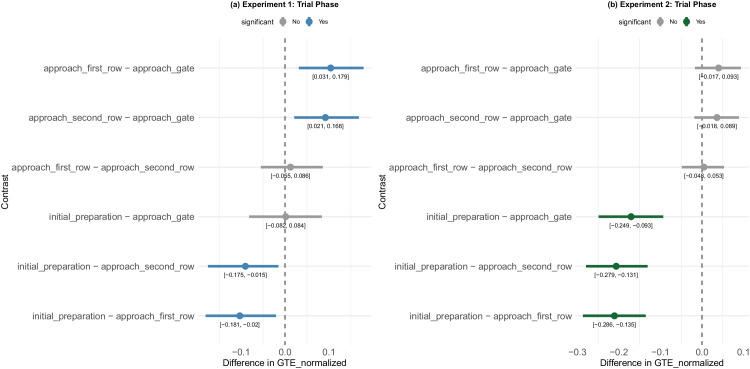
Pairwise comparisons for gaze entropy. (a) Experiment 1: GTE contrasts. (b) Experiment 2: GTE contrasts.

Bayesian model comparison revealed evidence against the effect of trial phase effects in experiment 1 (BF_10_ = 0.21) and strong evidence for the effect in experiment 2 (BF_10_ = 71.68), but visual inspection of Figure 17 shows clear qualitative differences between the two bookend phases and the two navigational phases. Entropy was elevated during active navigation, with the values slightly higher in the approaching first row phase (orange) than in the approaching second row phase (violet). It is important to note that, when we ran the same analyses using data only from participants who had complete data for all four phases, the models could not be properly fitted due to too many divergent transitions. Post hoc analyses showed that, despite evidence against the main effect in experiment 1, some of the pairwise comparisons between the bookend and navigational phases were statistically significant (see Tables 11, 12, and Figure 29 in Appendix A).

#### K-coefficient analysis

To obtain a more more complete picture of gaze allocation, we supplemented the GTE analysis with the K-coefficient, which is calculated by subtracting standardized saccade amplitude from standardized fixation duration:

(1) $$K=\frac{d_{i}-\mu_{d}}{\sigma_{d}}-\frac{a_{i}-\mu_{a}}{\sigma_{a}},$$

where *d*_*i*_ is fixation duration, *a*_*i*_ is subsequent saccade amplitude, μ_*d*_ and μ_*a*_ are the respective means, and σ_*d*_ and σ_*a*_ are the standard deviations. Values above zero indicate focal attention (longer fixations, shorter saccades), whereas values below zero indicate ambient attention (shorter fixations, longer saccades).

**Figure 30.**
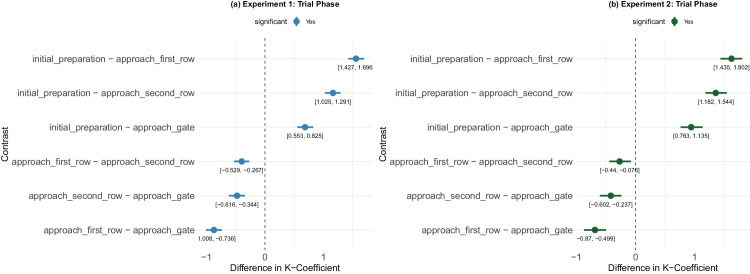
Pairwise comparisons for K-coefficient. (a) Experiment 1: K-coefficient contrasts. (b) Experiment 2: K-coefficient contrasts.

Unlike in the GTE analyses, Bayesian model comparisons showed strong effect of trial phase on the K-coefficient in both experiments (experiment 1: BF_10_ = 7.76 × 10^27^; experiment 2: BF_10_ = 2.59 × 10^25^). Post hoc analyses confirmed that all pairwise comparisons were significant because none of the credible intervals overlapped zero (see Tables 13, 14; Figure 30).

Taken together, these analyses revealed a coherent and stable pattern of information-seeking over the course of a trial. Phases with the highest GTE showed the most ambient gaze, a result that gives more weight to the claim that there was a qualitative difference between the navigational and non-navigational phases. It appears that our participants were heavily focused on the gate indicator in the initial preparation phase, followed by highly entropic/ambient gaze while navigating, which recovered to more stable again during the approaching gate phase. These results agree with our task structure, and provide some insight about participants’ strategies. One notable finding is the discrepancy in the precision of the two methods. Although the mean values of GTE showed a trend similar to the K-coefficient, the results of the analysis were not conclusive and the error bars wide. This may reflect that GTE was designed for analysis of stationary eyetracking data, while the K-coefficient is more generally applicable, and gives results that appear to be more precise and replicable, even when applied to data collected during a dynamic task.

### Fixations, saccades, and smooth pursuit

In light of the salience of moving agents in the SW task, we next searched for a method of classifying eye movements that included smooth pursuit as well as fixations and saccades. In recent years, hidden Markov models (HMMs) have emerged as an effective tool for eye movement classification (Kasneci et al., 2014). We used the gazeHMM R package (Lüken, Kucharskỳ, & Visser, 2022) to classify our eye data into 4 states: fixations, saccades, post-saccadic oscillations and smooth pursuit.

#### Fixations

Fixation analyses revealed stable patterns across both experiments. Bayesian model comparison revealed a strong effect of trial phase on fixation duration in both experiments (BF_10_ = 8.47 × 10^24^ in experiment 1 and BF_10_ = 3.54 × 10^114^ in experiment 2). The initial preparation phase had by far the longest mean fixation durations, reflecting participants’ focal attention on the gate indicator sign. Mean fixation times in subsequent phases were less than one-half those in initial preparation, as participants gathered information from a greater variety of sources. The number of fixations per phase reveals another interesting pattern: the approaching second row phase contained close to three fixations per phase, whereas the preceding approaching first row had only about one fixation. This finding agrees with the result reported above that, while the approaching first row phase had high gaze entropy, the approaching second row phase had more structured and predictable gaze allocation. Visual inspection of a random subset of trials via the replay system confirmed that during the initial preparation phase, participants mostly concentrated their gaze on the gate indicator, sometimes rapidly saccading to the timer for a quick check. Although the mean fixation duration during initial preparation was still elevated compared with other phases, the replay system suggests that the fixations on the gate indicator might have been much longer than the mean value, which was brought down by rapid fixations on the timer. To address this issue, we checked the median fixation duration, which was roughly 100 ms shorter than the mean in both experiments. This finding suggests that the distribution is positively skewed, with long fixations in the right tail. For all the other phases the two values were much closer to each other.

#### Saccades

Some additional insight is provided by the analysis of saccades. Bayesian model comparison revealed strong effect of trial phase on saccade amplitude in both experiments (BF_10_ = 3.82 × 10^7^ in experiment 1 and BF_10_ = 1.47 × 10^15^). There were fewer saccades in initial preparation than the next two phases, although more than in the final phase, where saccades were rare. Moreover, the mean amplitude of saccades (in degrees of visual angle) in the initial preparation phase was smaller than in subsequent phases These patterns, in conjunction with some of the results presented in earlier sections, suggest that while participants awaited the target gate number, they were reluctant to let their gaze stray from the gate indicator sign. This result suggests that participants deployed their gaze strategically to carry out the task efficiently.

#### Smooth pursuit

In a dynamic task, one of the most potentially revealing gaze patterns is smooth pursuit (Kowler et al., 2019), an eye movement that indicates tracking of moving agents and dynamic elements. To validate our use of gazeHMM (Lüken, Kucharskỳ, & Visser, 2022), we analyzed the resulting eye movement patterns in several single trials. As an example, Figure 21 shows gaze classification over the course of a typical trial. Especially during the approaching second row phase, the plot shows periods of smooth pursuit (in violet), followed by high velocity saccades (red), post saccadic oscillations (green), more episodes of smooth pursuit, and fixations (blue). Based on the task design and the visual scanning efficiency analyses (Local/global gaze analyses), we would expect noisy and unstructured patterns at early *z*-positions (before crossing the first row), followed by periods of smooth pursuit when tracking the agents, which themselves are broken up by saccades. Thus, although the data are somewhat noisy, this pattern seems meaningful and plausible in light of the task structure.

Bayesian model comparison revealed a strong effect of trial phase on smooth pursuit duration in both experiments (BF_10_ = 1.10 × 10^9^ for experiment 1 and BF_10_ = 1.07 × 10^7^ for experiment 2). One notable pattern is that overall, there were more episodes of smooth pursuit in approaching second row relative to approaching first row. This agrees with the K-coefficient measure, which showed elevated ambient gaze in the approaching first row phase, notwithstanding a roughly equal number of saccades. This finding is consistent with participants being able to focus their gaze more efficiently when they had more time to do so (recall that approaching second row is a longer interval than approaching first row owing to the greater distance before the row).

Taken together, our analyses of eye movements tell a coherent story about how gaze is allocated over the course of each trial. During the initial preparation phase, participants fixate mostly on the gate indicator sign, while simultaneously gathering information about the environment via a relatively scattered pattern of low-amplitude saccades. Once the participant begins moving, gaze becomes more disorganized and dynamic, possibly because the first row of walkers is very close, and thus required very dispersed gaze to take in its entirety. (Recall that the field of vision in HMDs is reduced.) In approaching second row, the distance to the crossing is almost twice as large, giving the participant more time to organize and focus their gaze on the second row of walkers. Finally, during the approaching gate phase, participants look around much less, and mostly fixated on the target gate and the timer.

#### Local/global gaze analyses

Finally, we analyzed gaze patterns as a function of the hierarchical structure of the task (see Figure 2). As explained elsewhere in this article, we classified gaze locations during each phase as local, global, task related, or miscellaneous depending on how they related to the planning structure of the task. To examine the relationship between attention allocation and navigation performance at a fine temporal scale, we divided each trial phase into four temporal segments (start, early, middle, late), leading to 16 mini-phases (each lasting less than 1 second). Figure 23 shows the proportion of attention types across trial mini-phases.

The distribution of gaze allocations over trial phases showed a systematic shift that accords with the conclusions presented above. This analysis helps to clarify how participants divided their attention along the subgoal hierarchy. In both experiments combined, local looks were the most common type (around 36%), followed by the task-related (∼29%), miscellaneous (∼25%) and global (∼10%). As shown in Figure 23, in both experiments, during the initial preparation phase when participants were waiting to learn which gate to proceed to, they focused on the gate indicator (task-related), largely ignoring the agents entering the scene. In the approaching first row phase, after the target gate number was revealed, gaze lingered at the gate indicator for a few hundred milliseconds, and then became highly local while passing the first row. Looks during the approaching second row phase included a mixture of all four attention types. This steady pattern was most likely the result of additional time/distance available when crossing the second row, relative to the crossing of the first row. Most of the looks during the approaching gate phase were local looks toward the ultimate goal—the exit gate. However, there were some differences between experiments 1 and 2: participants in experiment 2 looked at the timer more, presumably because they were under greater time pressure than participants in experiment 1.

In summary, these figures depict how, over the course of a trial, participants dynamically changed how they deployed their gaze to reflect the subtasks they faced.

### Correlation between gaze and movement direction

Next we examined the relationship between gaze direction and participants’ heading. Unfortunately, the HMD used in these experiments did not record the headset orientation (more recent versions do). As the best available proxy, we calculated the direction of the participant’s bodily motion, and compared it with gaze direction. More specifically, we calculated the angle between the movement direction vector and the gaze direction vector, with 0° indicating alignment, positive/negative values indicating right/left shift from the direction of the movement, and ±180° indicating a look backward. Figure 24 shows symmetrical distributions centered around 0°, with substantial density across the entire range, which indicates that participants sometimes looked backwards.

Inspection of the joint distribution of alignment and body velocity (Figure 24) shows that high levels of misalignment were exclusively seen in slow and stationary movements, and arose mostly during the initial preparation phase. This is most likely the result of head movements in support of rapid environment scanning while stationary, which led to high misalignment values. For a more granular breakdown, see Figure 32 in Appendix A.

### Measuring cognitive load via the low/high index of pupillary activity (LHIPA)

Finally, we analyzed cognitive load, the quantity of mental processing required at a given moment of behavior. To measure cognitive load, we used the LHIPA, a wavelet-based measure which is derived from pupil diameter oscillations and is designed to control for variation in pupil size attributable to luminance changes (Duchowski et al., 2020). LHIPA has been shown to have an inverse relationship with cognitive load (with higher values indicating lower cognitive load), and is regarded as particularly suitable for dynamic VR environments because it is invariant to luminance (Pan et al., 2024; Guillon et al., 2016).

**Figure 31.**
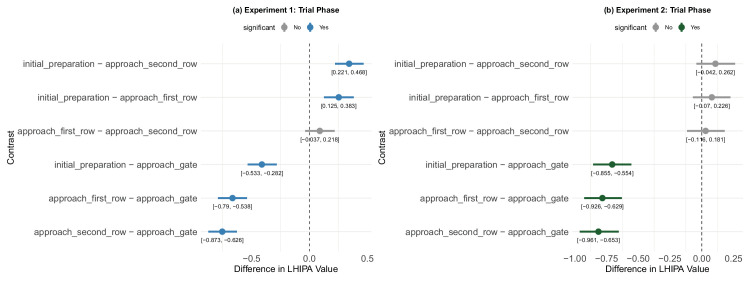
Pairwise comparisons for LHIPA. (a) Experiment 1: LHIPA contrasts. (b) Experiment 2: LHIPA contrasts.

**Figure 32.**
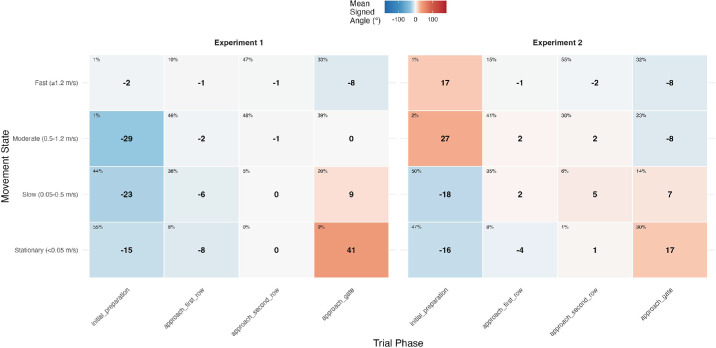
Exact distribution of mean alignment angles and speeds.

There was a strong effect of trial phase on the LHIPA value in both experiments (experiment 1 BF_10_ = 7.15 × 10^15^; experiment 2 BF_10_ = 5.63 × 10^14^). The apparent trend shows that the cognitive load increased through the first three phases, followed by a steep relief in the final phase when all the problem solving has been completed. Visual inspection of the plots revealed an interesting difference between experiments 1 and 2. While in experiment 1 the cognitive load during the initial preparation phase was lower than that in experiment 2, it appears to be somewhat higher during both the navigational phases. There was a more robust relief of cognitive load in experiment 2, corroborating the conclusion that experiment 2 required a more sustained cognitive load in the earlier part of each trial. Interestingly, the trend in experiment 2. was much steadier throughout the first three phases. Taken as a whole, this finding suggests that the overt manipulation of the deadline in experiment 2 induced participants to check the timer at the beginning of the trial. Although this initially increased their cognitive load, it made it easier to carry in subsequent phases. For post hoc analyses, see Tables 15, 16 and Figure 31.

## Summary of results

In this section, we briefly summarize the results, highlighting the main findings. For ease of exposition, we start with data about the participants’ movement trajectories, then discuss eye movements, and finally the interaction between them.

### Movement trajectories

In both experiment 1 (Figure 11) and experiment 2 (Figure 12), participants’ overall trajectories were sensitive to both static objects (standers, sitters, and benches) and moving obstacles (walkers). Furthermore, participants’ trajectories in experiment 1 showed that not all static obstacles are treated the same. Consistent with our previous studies involving static agents (De Stefani, 2023), participants gave less clearance to empty benches than occupied ones, apparently modifying their paths to respect the agents’ personal space (Figure 11). In experiment 2, the sitters were always present, but participants gave them clearance in inverse proportion to the time pressure (Figure 12b), suggesting that their strategy was to give the sitters as wide a berth as time would allow. Both of these findings indicate that participants (implicitly) made compromises between taking the most efficient route and adhering to social norms. These tradeoffs are particularly remarkable because they at least partly override the well-established tendency in human locomotion to minimize metabolic costs (Bertram, 2005). For example, both participants’ tendency to deviate from the midline in the presence of sitters, and the reduced sensitivity to time constraints in experiment 2, suggest that metabolic costs were not the participants’ sole priority. Indeed, the elevated cognitive load that we observed during the two navigational phases itself represents a metabolic cost (Jamadar, Behler, Deery, & Breakspear, 2025) that the participants expended to maximize adherence to social constraints.

In addition to these global effects, there are several lines of evidence that suggest that participants modified their paths locally in response to immediate input. Most obviously, participants never collided with moving agents, but instead invariably passed between them, decelerating to achieve the correct timing (Figures 9 and 10). Moreover, participants gravitated toward the middle of each inter-agent gap, except when under the most extreme time pressure (Figure 14). Note that, because the moving agents all walked at constant speed, their positions were technically predictable from the outset. However, our participants’ strategy seems to have been to adjust their path and speed on the fly in response to dynamic visual input.

### Eye movements

The interplay between goals and subgoals was especially clear in the eye movement data. Overall, task-related and local gaze accounted for around 55% of all gaze, with about 12% designated global (Figure 23). We observed clear variation of gaze allocation depending on the attention type, with consistent patterns in both experiments. Granular analysis revealed shifts in attention allocation on a sub-second scale, which can be plausibly attributed to the sub-task currently undertaken by the participant.

Generally, at the beginning of each trial, participants’ eye movements were directed focally on the gate indicator, but once they began to move, their attention was distributed across a variety of objects and information sources, as showed by elevated saccade counts during the navigational phases (Figure 20). Participants’ gaze was most dispersed when they were shifting from the preparatory phase to actively moving (Figures 18, 23). As navigation progressed, gaze became more organized (Figures 17, 23) and less ambient (Figure 18). Smooth pursuit peaked during the approaching second row phase (Figure 22), showing that at that point participants were well in control of which targets to track in the environment. This rapid movement of attention among different types of targets entailed a higher cognitive load, as evidenced by the pupillometry findings (Figure 25). These data show that active navigation required significantly more mental effort than the other two phases. Approaching gate showed cognitive relief, combined with focal and organized gaze, with more looks to the timer in experiment 2 where the deadline was more salient (Figure 23).

## Discussion

This study aimed to advance the state of the art in the analysis of complex and naturalistic behavioral tasks, and also to provide some novel insights about SW. Even though the task was fairly simple (walk to a target location), the social and physical constraints that the wayfinder had to satisfy led to a complex series of local decisions in service of the larger goal.

The ever-changing visual input made it necessary for the visual system to continuously reevaluate which elements in the environment required further scrutiny, and potentially a behavioral response such as acceleration, direction change, or direct gaze.

Our overarching conclusion is that participants’ planning and decision-making was hierarchical, involving both a global goal (arriving punctually at the right gate) and a series of constantly changing subgoals (avoiding obstacles). This hierarchical structure was evident in both the participants’ movement trajectories and in their eye movements. From the outset, participants planned trajectories that obeyed a combination of physical and social constraints. But they also modified these trajectories in light of the obstacles they encountered, thereby threading the needle and passing through the crowd in an efficient, collision-free manner. Participants’ eye movements played a crucial role in making this careful coordination possible, rapidly alternating among targets that pertained to the overall goal (such as the timer), a future goal (such as a walker in the next row), or a more immediate goal (such as an agent they were about to pass). This carefully coordinated progression of decisions unfolded in the course of only a few seconds, and was almost always successful. It is striking that our participants successfully carried out the task after only a brief tutorial, presumably harnessing their experience as social wayfinders in real life to guide them in a novel VR environment.

Overall, our results support Land et al. (1999)’s contention that almost all eye movements in natural tasks relate directly to the task at hand. Participants looked mostly at moving agents (who they needed to track in order to avoid collisions). However Land et al. (1999)’s further conclusion that gaze is usually directed at manipulated objects is insufficiently general. In our task, the relevant target was not an object under the observer’s control at all, but rather an object or location in the environment or an independently moving person. This further highlights the flexibility of the system, in which eye movements are dynamically deployed to whatever features of the environment relate to the observer’s goals.

As mentioned, although a few studies have recorded eye movements during naturalistic VR tasks (Pelz & Canosa, 2001; Rothkopf, Ballard, & Hayhoe, 2007; Rothkopf & Ballard, 2009; Zhang et al., 2018), or with behaviorally relevant moving obstacles (Raimbaud et al., 2023), we believe that ours is the first to present participants with such a complex task, requiring simultaneous information-gathering and locomotion under a looming deadline. This task structure greatly complicates both the problem the participant must solve and the analysis of the resulting data. Indeed, notwithstanding the analytical complexity that we emphasized above, SW is a very natural task to which our subjects came already extensively trained by everyday experience. However, the way eye movements are deployed to support such a task is substantially unknown. Our study takes a step toward understanding how people carry out complex dynamic tasks, including the eye movements that support them. We hope this paper demonstrates the feasibility of carrying out this kind of study, potentially in even more complex behavioral tasks.

### Conclusions about SW

Our results provide some novel conclusions about SW. First, as suggested by previous studies involving static obstacles (DeStefani et al., 2023), wayfinders choose a path that satisfies a combination of social and non-social cues (Chen, McNamara, Kelly, & Wolbers, 2017; Newman, Qi, Mou, & McNamara, 2023) and constraints. Non-social constraints include the desire to arrive at the target location while avoiding collisions with physical obstacles such as walls and benches. Specifically, social constraints are more subtle, and include respect for other humans’ proxemic preferences (Hayduk, 1983), such as avoiding passing close in front of them (Sanz et al., 2015; DeStefani, Stromswold, & Feldman, 2022). But in our task, as in many real-life situations, it was not usually possible to avoid passing in front of people. Thus, our participants had to plan carefully how to pass through the crowd, usually passing through the middle of inter-agent gaps unless time pressure forced them to sacrifice social graces for efficiency (while still respecting personal space to the extent possible). The great care participants took to observe these social constraints is demonstrated by the fact that when approaching the first row, gaze briefly became more local relative to the rest of the trial. This finding suggests that participants allocated extra resources to make sure they did not collide with the two agents. This effect was not as pronounced in the second row, presumably because participants had enough time to identify the target crossing point using a more balanced allocation of gaze (Hu, Yoon, Pavlovic, Faloutsos, & Kapadia, 2020).

Finally, we note that our results provide further validation for the use of VR to study real-world tasks like SW, especially now that the requisite hardware, software, and analytical techniques are readily available. These tools make it possible to paint a detailed and reliable picture of the cognitive processes at play in naturalistic tasks. In our debrief survey, participants answered a series of questions concerning their VR experience, probing their perception of the environment as well as their behavioral patterns. A substantial majority reported that the train station felt “realistic,” and that they behaved and reacted to the agents the same way they would in real life. Indeed, subjects never broke the rules of physics, for example, by walking through benches or other agents. This pattern was observed in real time by the experimenter, and also confirmed later by analysis of the participants’ data, which never included outlier trajectories suggesting physically impossible shortcuts.

### Connections to other fields

In addition to the psychological findings in the domain of human SW, we believe our findings have practical implications, in a manner similar to the field of human factors (Montello & Sas, 2006). One example is robot navigation, which has been criticized for being insufficiently social (Gao & Huang, 2022). Arguably, the main obstacle to human-like navigation in robots is insufficient understanding of SW in humans (Möller, Furnari, Battiato, Härmä, & Farinella, 2021). The participants in our experiments exhibited a number of behaviors not reflected in contemporary robotic navigation algorithms, such as the carefully calibrated manner in which they passed near agents (Greenberg, Gonzalez-Bravo, Yi, & Feldman, 2025a; Greenberg et al., 2025b). Our hope is that future robots will make way for people in much the same fluid and socially graceful way that our participants did for the (virtual) humans in our (virtual) train station.

Additionally, our focus on crowded indoor spaces connects to important public safety considerations (Miao, 2024). To allow for safe and comfortable experiences, architects and urban designers need accurate models of human pedestrian movement, which requires careful empirical study of human navigation. A more complete understanding of how humans navigate through populated spaces will make it possible for buildings and public spaces to be designed in a manner that more accurately reflects the way people actually move through them.

## Conclusions

In this article, we presented two SW related experiments, which allowed us to draw some novel conclusions about SW, and about the hierarchical nature of real-time decision making and eye movements in naturalistic tasks. We used a novel VR paradigm, in which participants physically moved through a virtual environment while wearing a HMD. This methodology provides detailed data about both participants’ locomotion and their eye movements, which together allowed us to create a comprehensive picture of decision-making in a navigational task.

The studies described above demonstrate practical procedures and analytical techniques for carrying out VR studies with eye-tracking, which we hope can be extended to study other cognitive and social phenomena. In our own future work, we hope to introduce more naturally moving and intelligent agents into the VR environment, further amplifying the ecological validity and immersion of the paradigm. We hope that other laboratories will find these techniques useful, and will use them to study a range of topics in psychology and cognitive science that are difficult to study in real life.

## Footnotes

1 There is a direct train from New Brunswick, NJ, to New York City, which Rutgers University students often ride.

## Appendix A

**Table 5.** Pairwise comparisons for success by sitter position effects (experiment 1).

Experiment	Contrast	Diff	95% CI	Sig
1	Sitters on both sides - sitters on gate side	0.010	[0 to 0.019]	No
1	Sitters on both sides - sitters opposite gate	0.003	[−0.006 to 0.013]	No
1	Sitters on gate side - sitters opposite gate	−0.007	[−0.016 to 0.003]	No

**Table 6.** Pairwise comparisons for success by time condition effects (experiment 2).

Experiment	Contrast	Diff	95% CI	Sig
2	time_condition08 - time_condition10	−0.179	[−0.264 to −0.095]	Yes
2	time_condition08 - time_condition12	−0.229	[−0.314 to −0.142]	Yes
2	time_condition10 - time_condition12	−0.050	[−0.136 to 0.033]	No

**Table 7.** Pairwise comparisons for trial duration by sitter position (experiment 1).

1	Sitters on both sides - sitters on gate side	−0.06	[−0.17 to 0.03]	No
1	Sitters on both sides - sitters opposite gate	0.15	[0.05 to 0.25]	Yes
1	Sitters on gate side - sitters opposite gate	0.21	[0.11 to 0.31]	Yes

**Table 8.** Pairwise comparisons for trial duration by time condition (experiment 2).

Experiment	Contrast	Diff (s)	95% CI	Sig
2	time_condition08 - time_condition10	−0.36	[−0.55 to −0.16]	Yes
2	time_condition08 - time_condition12	−0.69	[−0.88 to −0.49]	Yes
2	time_condition10 - time_condition12	−0.33	[−0.53 to −0.14]	Yes

**Table 9.** Pairwise comparisons for distance from midline by sitter position (experiment 1).

Experiment	Contrast	Diff	95% CI	Sig
1	Sitters on both sides - sitters on gate side	0.054 m	[0.037 to 0.071]	Yes
1	Sitters on both sides - sitters opposite gate	0.122 m	[0.105 to 0.139]	Yes
1	Sitters on gate side - sitters opposite gate	0.068 m	[0.052 to 0.085]	Yes

**Table 10.** Pairwise comparisons for distance from midline by time condition (experiment 2).

Experiment	Contrast	Diff	95% CI	Sig
2	Deadline 08 - deadline 10	−0.016 m	[−0.031 to −0.001]	Yes
2	Deadline 08 - deadline 12	−0.028 m	[−0.044 to −0.014]	Yes
2	Deadline 10 - deadline 12	−0.012 m	[−0.027 to 0.003]	No

**Table 11.** Pairwise comparisons for trial phase effects (GTE normalized - experiment 1).

Experiment	Contrast	Diff	95% CI	Sig
1	Initial preparation - approaching first row	−0.103	[−0.181 to −0.02]	Yes
1	Initial preparation - approaching second row	−0.090	[−0.175 to −0.015]	Yes
1	Initial preparation - approaching gate	0.002	[−0.082 to 0.084]	No
1	Approaching first row - approaching second row	0.013	[−0.055 to 0.086]	No
1	Approaching first row - approaching gate	0.104	[0.031 to 0.179]	Yes
1	Approaching second row - approaching gate	0.092	[0.021 to 0.168]	Yes

**Table 12.** Pairwise comparisons for trial phase effects (GTE normalized - experiment 2).

Experiment	Contrast	Diff	95% CI	Sig
2	Initial preparation - approaching first row	−0.211	[−0.286 to −0.135]	Yes
2	Initial preparation - approaching second row	−0.206	[−0.279 to −0.131]	Yes
2	Initial preparation - approaching gate	−0.171	[−0.249 to −0.093]	Yes
2	Approaching first row - approaching second row	0.005	[−0.048 to 0.053]	No
2	Approaching first row - approaching gate	0.040	[−0.017 to 0.093]	No
2	Approaching second row - approaching gate	0.036	[−0.018 to 0.089]	No

**Table 13.** Pairwise comparisons for trial phase effects (K-coefficient - experiment 1).

Experiment	Contrast	Diff	95% CI	Sig
1	Initial preparation - approaching first row	1.5611495	[1.427 to 1.694]	Yes
1	Initial preparation - approaching second row	1.1513706	[1.02 to 1.287]	Yes
1	Initial preparation - approaching gate	0.6856037	[0.547 to 0.817]	Yes
1	Approaching first row - approaching second row	−0.4095721	[−0.545 to −0.275]	Yes
1	Approaching first row - approaching gate	−0.8767164	[−1.014 to −0.737]	Yes
1	Approaching second row - approaching gate	−0.4660973	[−0.603 to −0.333]	Yes

**Table 14.** Pairwise comparisons for trial phase effects (K-coefficient - experiment 2).

Experiment	Contrast	Diff	95% CI	Sig
2	Initial preparation - approaching first row	1.7105291	[1.535 to 1.877]	Yes
2	Initial preparation - approaching second row	1.3857417	[1.212 to 1.562]	Yes
2	Initial preparation - approaching gate	0.9878661	[0.822 to 1.162]	Yes
2	Approaching first row - approaching second row	−0.3235997	[−0.498 to −0.158]	Yes
2	Approaching first row - approaching gate	−0.7230805	[−0.893 to −0.556]	Yes
2	Approaching second row - approaching gate	−0.3980844	[−0.564 to −0.225]	Yes

**Table 15.** Pairwise contrasts for trial phase effects (LHIPA - experiment 1).

Experiment	Contrast	Diff	95% CI	Sig
1	Initial preparation - approaching first row	0.2522468	[0.123 to 0.375]	Yes
1	Initial preparation - approaching second row	0.3407764	[0.217 to 0.469]	Yes
1	Initial preparation - approaching gate	−0.4166483	[−0.54 to −0.289]	Yes
1	Approaching first row - approaching second row	0.0876508	[−0.028 to 0.219]	No
1	Approaching first row - approaching gate	−0.6687321	[−0.786 to −0.538]	Yes
1	Approaching second row - approaching gate	−0.7567402	[−0.876 to −0.626]	Yes

**Table 16.** Pairwise contrasts for trial phase effects (LHIPA - experiment 2).

Experiment	Contrast	Diff	95% CI	Sig
2	Initial preparation - approaching first row	0.0800445	[−0.068 to 0.23]	No
2	Initial preparation - approaching second row	0.1065271	[−0.044 to 0.256]	No
2	Initial preparation - approaching gate	−0.7074436	[−0.852 to −0.553]	Yes
2	Approaching first row - approaching second row	0.0248840	[−0.126 to 0.178]	No
2	Approaching first row - approaching gate	−0.7863454	[−0.94 to −0.644]	Yes
2	Approaching second row - approaching gate	−0.8123418	[−0.959 to −0.658]	Yes
